# Multimodal optical contrast agents as new tools for monitoring and tuning nanoemulsion internalisation into cancer cells. From live cell imaging to *in vivo* imaging of tumours†

**DOI:** 10.1039/c9na00710e

**Published:** 2020-02-27

**Authors:** Geoffrey Prévot, Talia Bsaibess, Jonathan Daniel, Coralie Genevois, Guillaume Clermont, Isabelle Sasaki, Sebastien Marais, Franck Couillaud, Sylvie Crauste-Manciet, Mireille Blanchard-Desce

**Affiliations:** Univ. Bordeaux, Institut des Sciences Moléculaires (CNRS UMR 5255) 33405 Talence France mireille.blanchard-desce@u-bordeaux.fr; Univ. Bordeaux, ARNA Laboratory, Team ChemBioPharm, U1212 INSERM – UMR 5320 CNRS 146 Rue Léo Saignat 33076 Bordeaux Cedex France; Univ. Bordeaux, Molecular Imaging and Innovative Therapies (IMOTION), EA7435 Bordeaux 33000 France; Bordeaux Imaging Center, UMS 3420 CNRS - Univ. Bordeaux, US4 Inserm 33000 Bordeaux France; Pharmaceutical Technology Department, Bordeaux University Hospital Bordeaux France

## Abstract

Tailor-made NIR emitting dyes were designed as multimodal optical probes. These asymmetric amphiphilic compounds show combined intense absorption in the visible region, NIR fluorescence emission, high two-photon absorption in the NIR (with the maximum located around 1000 nm) as well as large Stokes' shift values and second-harmonic generation ability. Thanks to their structure, high loading into nanoemulsions (NEs) could be achieved leading to very high one- and two-photon brightness. These dyes were demonstrated to act as multimodal contrast agents able to generate different optical modalities of interest for bioimaging. Indeed, the uptake and carrier behaviour of the dye-loaded NEs into cancer cells could be monitored by simultaneous two-photon fluorescence and second-harmonic generation optical imaging. Multimodal imaging provided deep insight into the mechanism and kinetics of dye internalisation. Quite interestingly, the nature of the dyes was also found to influence both the kinetics of endocytosis and the internalisation pathways in glioblastoma cancer cells. By modulating the charge distribution within the dyes, the NEs can be tuned to escape lysosomes and enter the mitochondria. Moreover, surface functionalization with PEG macromolecules was realized to yield stealth NIRF-NEs which could be used for *in vivo* NIRF imaging of subcutaneous tumours in mice.

## Abstract 2

Tailor-made NIR emitting dyes were designed as multimodal optical probes.

## Graphical abstract

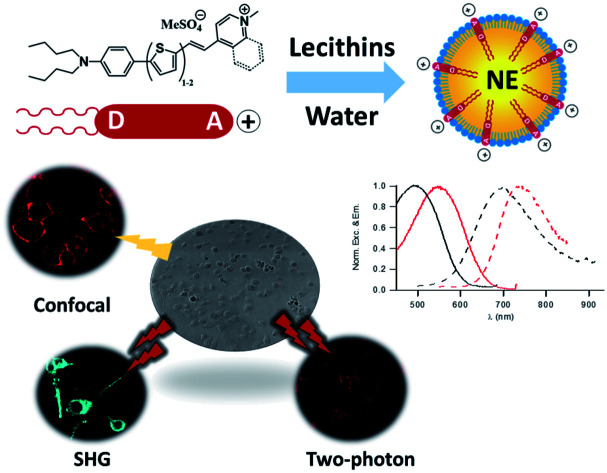

## Introduction

Non-invasive molecular imaging has great potential for human clinical use both for diagnosis and treatment. Among the diverse imaging techniques available, near-infrared fluorescence (NIRF) imaging is emerging as a promising tool to revolutionize the medical field, providing insights into cellular and molecular events under both physiological and pathological conditions. NIRF is highly sensitive, quantitative, cost-effective and easy to operate. It also represents a powerful intraoperative tool, allowing tumour visualization^1^ and subsequently highly precise tumour resection with minimal peritumoral damage.^2^ However, to become a clinical reality and a routine technology, optimized NIR fluorescent contrast agents are still needed. Several types of fluorescent tracers have been developed including organic dyes, molecular-based nanoparticles,^3^ dye-loaded organic or inorganic nanoparticles and fluorescent quantum dots (QDs).^4^ Among them, fluorescent QDs have become extremely popular in biological and biophysical studies thanks to their high brightness and photostability. Yet, the brightest ones raise concerns in terms of safety as both acute and chronic toxicity have been demonstrated.^5^ In contrast, NIRF dyes may show improved biocompatibility as is the case for indocyanine green and methylene blue which are approved fluorescent tracers. Still, these dyes present several restrictions including limited brightness in water, concentration-dependent aggregation and non-specific binding to plasma proteins.^6^

Nanoemulsions (NEs) are some of the best candidates among the large variety of nanocarriers currently investigated. Oil-in-water (o/w) NEs are dispersed systems of two non-miscible liquids, consisting of an oily system dispersed in an aqueous system, forming oil droplets of nanometer sizes (with diameter between 20 and 500 nm) stabilized by surfactants. NEs have been used in pharmaceutical therapy for more than 50 years, and according to the Nanotoxicological Classification System (NCS), NEs belong to the low risk class (*i.e.*, class 1) thanks to their size (over 100 nm) and their biodegradability.^7,8^ NEs as drug carriers are known to have numerous advantages over other nanosystems, including liposomes, such as long term physical stability and high loading capacity of lipophilic substances in the oily core and amphiphilic substances in the corona.^9^ Despite all these advantages, NEs have not yet been fully exploited in fluorescence imaging. For dual imaging applications, NEs have been designed as multimodal contrast agents for combined ultrasound and ^19^F MRI (perfluorocarbon NEs)^10^ or for combined MRI and X-ray imaging (iodine and magnetite NEs).^11^ Recently, Gianella *et al.*^12^ have developed multimodal NEs carrying iron oxide nanoparticles for MRI imaging and whose outside corona was labelled with lipid-PEG conjugated with Cy7 commercial dye for NIRF imaging. Particularly interesting systems, based on solid lipid nanoparticles (Lipidots),^13–15^ have also been developed. These nanoparticles could be loaded with various hydrophobic cyanine dyes (up to a few hundred molecules in the lipidot core), yielding a library of tunable lipidots emitting from the visible to the NIR. These bright and biocompatible NEs show low cytotoxicity and could be successfully used for *in vivo* NIR fluorescence (NIRF) imaging.^14,15^ However, to the best of our knowledge, the design of NEs as multimodal optical contrast agents by using a single contrast agent to generate different modalities has not been reported yet. Yet these different contrasts might offer interesting insight into the mechanisms of NE internalisation into cells.

In this context, our goal has been to achieve ultrabright fluorescent NEs as translational multimodal optical contrast agents that could be used both in *in vitro* multiphoton imaging and NIRF *in vivo* imaging. To this end, we designed new dedicated dyes that show far red to NIR1 fluorescence as well as large optical nonlinearities allowing their use for *multimodal linear and nonlinear optical (NLO) imaging.*

## Results and discussion

### Design and synthesis of the dyes for multimodal optical imaging

**Fig. 1:**
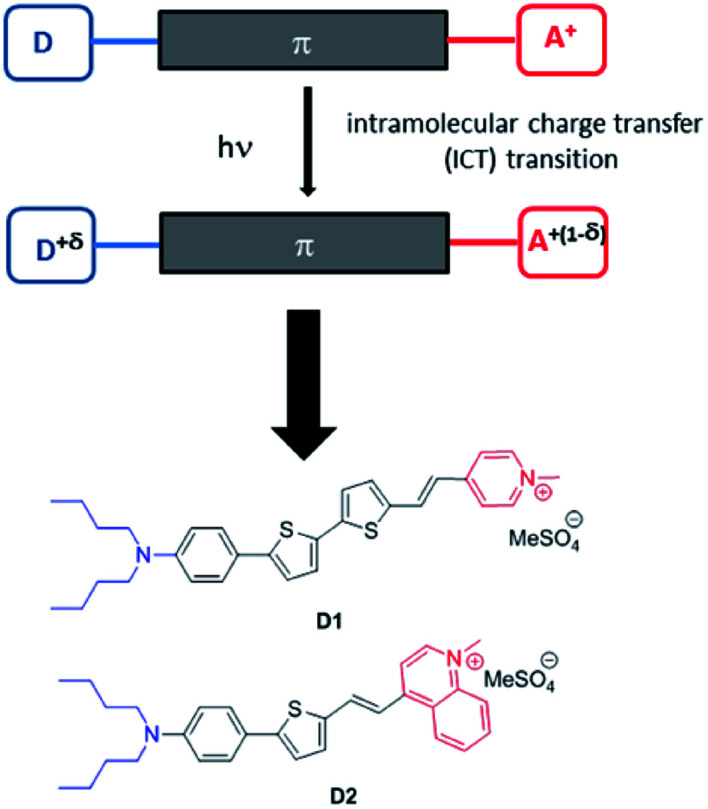
Design of the amphiphilic push–pull dyes D1 and D2. Upon excitation, a charge shift from the donor end-group (in blue) towards the acceptor end-group (in red) occurs leading to partial positive charge on the donating amino moiety and a corresponding decrease of positive charge on the pyridinium (or quinolinium) acceptor.

Two new fluorescent dyes have been designed to this aim. Dyes D1 and D2 are push–pull compounds which bear strong donor and acceptor end-groups as well as thiophene moieties in the π-conjugated system (Fig. 1). This type of hemicyanine dye is anticipated to show strong absorption in the visible region and fluorescence emission in the far-red to NIR region,^3,16^ in relation to an intramolecular charge transfer (ICT) transition from the donor to the acceptor upon excitation. For this same reason, such dyes were expected to display large two-photon absorption cross-sections (*σ*_2_) in the first biologically transparent window, *i.e.*, 700–1000 nm,^16,17^ as well as large quadratic hyperpolarizability β.^18^ Thanks to the presence of the two alkyl (*n*Bu) chains on the terminal nitrogen and of the positively charged opposite acceptor end-group (pyridinium or quinolinium moieties), the hemicyanine dyes show amphiphilic features which were meant to bestow them high affinity for the lipid corona of NEs. Due to their non-centrosymmetric structure and amphiphilic features (contrarily to cyanine dyes), these dyes were aimed to orientate in the lipid corona of NEs or the outer leaflet of lipidic membranes and generate second harmonic generation (SHG). In the past two decades, SHG microscopy has gained recognition in the bioimaging community.^19^ SHG microscopy has been used to image structural organization in proteins, especially collagen.^20^ Exogenous SHG membrane probes, including push–pull amphiphilic dyes, have also been developed as membrane dyes to probe membrane potentials.^21–26^

Hence amphiphilic push–pull dyes D1 and D2 were meant to act as multimodal optical contrast agents allowing (i) *in vitro* multiphoton imaging by simultaneous two-photon excited fluorescence (TPEF) and SHG microscopy,^23–30^ (ii) monitoring the fate of the encapsulated dyes in cancer cells at the sub-cellular level and (iii) *in vivo* imaging of tumours in small animals using NIRF.

**Scheme 1:**
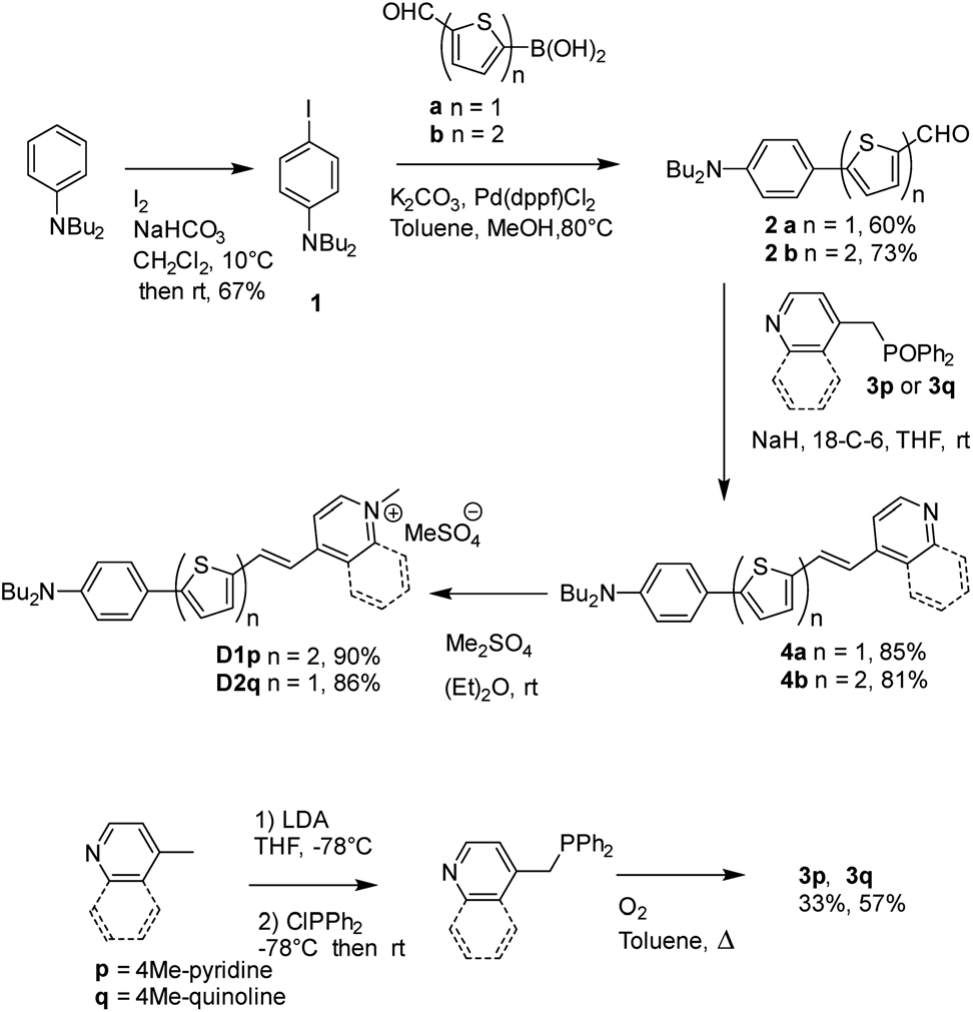
Synthesis of the D1 and D2 dyes.

The D1 and D2 dyes were synthesized as described in Scheme 1, starting from commercially available *N*,*N*-dibutyl-aniline. After iodination of *N*,*N*-dibutyl-aniline leading to 1 (67%), 1 was coupled to 5-formyl-2-thiopheneboronic acid or 5-formyl-2-2-bithiopheneboronic acid using a Suzuki–Miyaura-type reaction to yield aldehyde 2a or 2b (60% and 73%, respectively). The Wittig–Horner condensation between the aldehydes and the phosphine oxide 3p or 3q led to pyridine or quinoline 4a or 4b (85% and 81%, respectively) which further gave dyes D1 and D2 after methylation with dimethylsulfate. Reagents 3p and 3q were prepared in two steps from 4-picoline or 4-methylquinoline in a 33 or 57% total yield, respectively: the first step involved lithiation of 4-picoline or 4-methylquinoline with LDA followed by reaction with chlorodiphenylphosphine; in the second step, oxidation of the obtained intermediate phosphine with oxygen in refluxing toluene was carried out leading to compounds 3p and 3q.

**Scheme 2:**
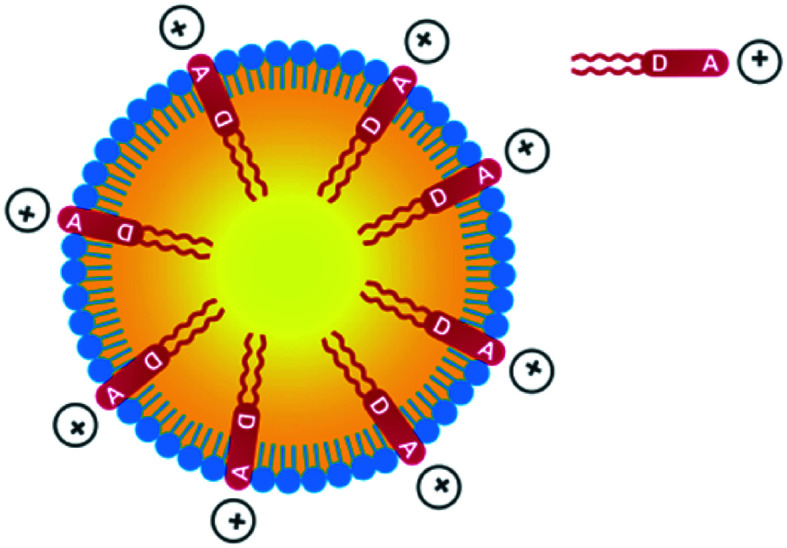
Incorporation of amphiphilic dyes D1 and D2 into NEs made from lecithins.

Thanks to the presence of the two *n*Bu alkyl chains, the D1 and D2 dyes are soluble in a range of organic solvents of interest for their encapsulation in NEs. In contrast, the dyes are non-soluble in water but are readily dispersed in water in the presence of surfactants. Their amphiphilic character was meant to favour high loading into the NE corona and preferential radial average orientation (Scheme 2), thus ensuring maximal absorption.

### Absorption and fluorescence properties of the D1 and D2 dyes

**Fig. 2:**
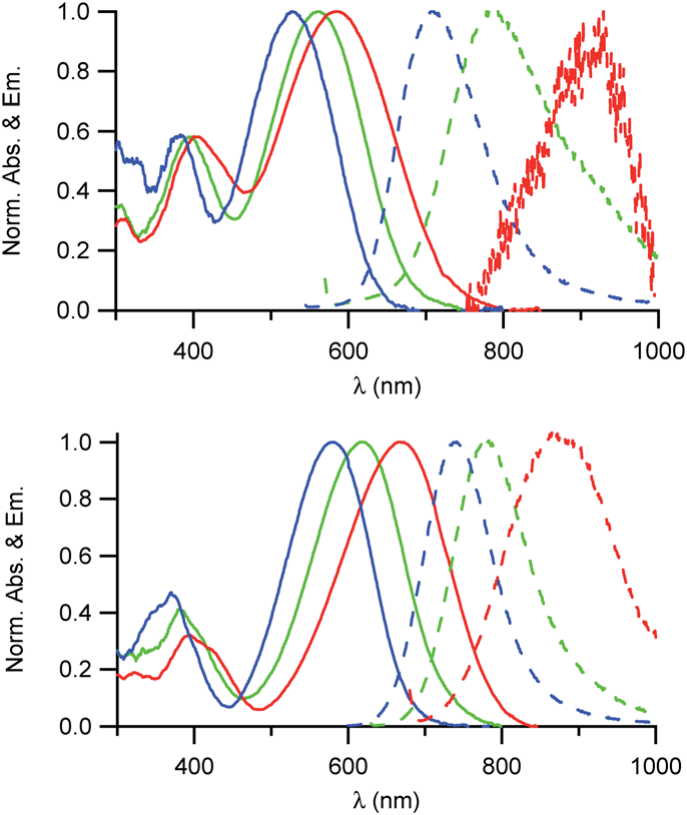
Absorption and emission spectra of the D1 (top) and D2 (bottom) dyes in solution in toluene (blue), CHCl_3_ (green) and CH_2_Cl_2_ (red).

As illustrated in Fig. 2, the D1 and D2 dyes show an intense low-energy absorption band in the visible region (with a maximum molar absorption coefficient of 3.7 × 10^4^ M^−1^ cm^−1^ and 3.9 × 10^4^ M^−1^ cm^−1^ in chloroform for D1 and D2, respectively) whose position strongly depends on the polarity of their environment. A marked red-shift of the low-energy absorption band is observed on going from low polarity solvents to medium polarity solvents (Table 1). This behaviour is consistent with a photo-induced intramolecular charge transfer (or charge shift) from the donor end-group to the acceptor end-group resulting in an increase of the dipole moment upon excitation (Fig. 1).

**Table 1:** Photophysical properties of the dyes

Dye	*λ* ^abs^_max_, nm	*ε*_max_, M^−1^ cm^−1^	*λ* ^em^_max_, nm	FWHM^a^, 10^3^ cm^−1^	*Φ*_f_	Stokes shift, 10^3^ cm^−1^
D1 in toluene	528		707	4.7	0.06^b^	4.8
D1 in CHCl_3_	560	3.70 × 10^4^	804	4.6	0.04^c^	5.4
D1 in CH_2_Cl_2_	585		915	5.2	<0.01	6.2
D1 in H_2_O^d^	523		717	4.6	0.11^b^	5.2
D2 in toluene	579		740	3.8	0.07^b^	3.8
D2 in CHCl_3_	616	3.95 × 10^4^	788	3.7	0.03^c^	3.55
D2 in CH_2_Cl_2_	668		876	3.6	<0.01	3.55
D2 in H_2_O^d^	584		722	3.8	0.14^b^	3.3

a Full width at half maximum.

b Standard: DCM in EtOH (*Φ*_f_ = 0.437).

c Standard: indocyanine green in DMSO (*Φ*_f_ = 0.11).

d Micellar water (SDS/butanol/water, 6 : 5 : 89 wt%).

The D1 and D2 dyes show fluorescence in organic solvents of low to medium polarity and emit in the far-red NIR1 region (Fig. 2). Similar to the absorption, the emission shows a marked positive solvatochromism in low to medium polarity organic solvents. A pronounced red-shift is observed on going from a low polarity solvent (*λ*^em^_max_ = 707 and 740 nm in toluene for D1 and D2, respectively) to a medium polarity solvent (*λ*^em^_max_ = 915 and 876 nm in dichloromethane). Again, this is consistent with a charge shift from the dialkylamino donor moiety to the pyridinium or quinolinium acceptor end-group upon excitation leading to larger dipole moment.

We also note that the fluorescence quantum yield decreases dramatically with increased polarity. As a consequence, dyes D1 and D2 show vanishing fluorescence in medium polarity environments and are not fluorescent in polar solvents. Due to the strong dependency of their absorption and fluorescence properties on the environment, the D1 and D2 dyes thus represent interesting environment probes. In addition, we note that the dyes show large Stokes shift values (Table 1), which is of interest for use in fluorescence imaging as it allows easier detection. This provides an advantage for fluorescence imaging compared to commonly used NIR fluorescent dyes such as cyanines which show very small Stokes shift values (typically 320 cm^−1^ for Cy5, 530 cm^−1^ for Cy5.5 and 400 cm^−1^ for Cy7).

We also note that dye D2 shows a red-shifted, more intense and narrower low-energy absorption band compared to D1 as well as smaller Stokes shift values (Table 1). This is further confirmed by the less marked solvatochromism of dye D2. This indicates that hemicyanine dye D2 has an electronic structure which is closer to the cyanine limit than dye D1. Hence the electronic distribution in the ground state of D2 is slightly different with small positive charge on the electron-donating dialkylamino end-group in the ground state. This difference in electronic distribution is consistent with the shortest conjugation path and the stronger acceptor end-group of D2 than that of D1.^17^ This difference in electronic distribution of the positive charge along the molecular axis is expected to lead to a more tilted orientation (or a more disordered organisation) into the lipid corona of the NE droplet and thus lower SHG activity.^26,30^

Thanks to their amphiphilic character, both dyes could be dissolved in water using SDS as a surfactant. We note that both absorption and emission spectra are similar to those measured in a low polarity solvent like toluene (Table 1 and Fig. S1†), indicating that the dyes are protected from the water environment. Furthermore, the fluorescence quantum yields are higher in a micellar environment thanks to the local viscosity which reduces non-radiative decay processes.

### Nonlinear optical properties of the D1 and D2 dyes

**Fig. 3:**
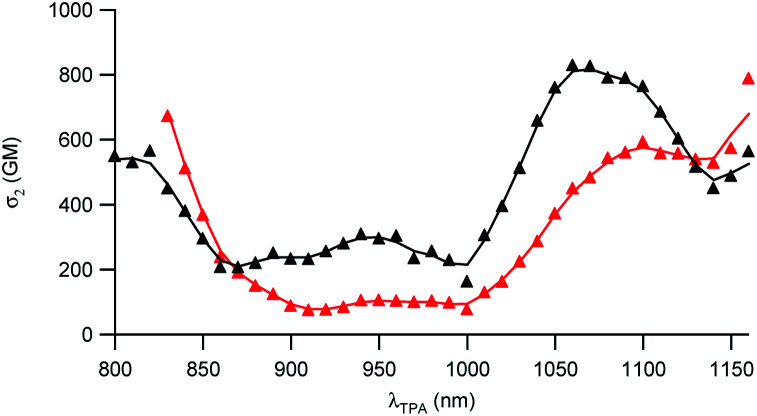
Two-photon absorption spectra of dyes D1 (black) and D2 (red).

The two-photon absorption (2PA) properties of the dyes were determined by conducting two-photon induced fluorescence (TPEF) experiments. The measurements were carried out in micellar water which ensures both good solubility and fluorescence of the dyes (*vide supra*). The TPEF measurements demonstrate that both dyes show large two-photon absorption responses in the 1050–1150 nm spectral region (Fig. 3). Both dyes show maximum 2PA cross-sections (*σ*_2_) of about 800 GM (830 GM at 1060 nm for dye D1 and 785 GM at 1160 nm for dye D2). The 2PA bands of dye D2 are red-shifted compared to those of dye D1 as noticed for the corresponding low-energy intense one-photon absorption band. Interestingly, an intense 2PA band is observed at higher energy which corresponds to a one-photon weakly allowed transition (Fig. S2†). As a result, both dyes also show a large 2PA close to 800 nm (565 GM at 820 nm for dye D1 and 672 GM at 830 nm for D2). Yet, we stress that when getting close to 800 nm, dye D2’s two-photon absorption overlaps with one-photon absorption as noted from the deviation to quadratic dependency of the fluorescence signal on the excitation intensity (Fig. S3†). A quadratic dependency is observed only at wavelengths longer than 830 nm. In contrast, dye D1 shows pure two-photon excitation at 800 nm (Fig. S4†).

These features indicate that dyes D1 and D2 can be used for two-photon excitation both at (i) long wavelengths (1050–1100 nm) allowing improved penetration and reduced background fluorescence in deep tissues and (ii) close to 800 nm which is the preferred wavelength to allow maximum output power when using popular lasers used in two-photon microscopy (*i.e.* Ti-sapphire laser). This makes dyes D1 and D2 of much interest for two-photon imaging by allowing both excitation and detection in the NIR1 region. We note that dye D2 shows larger 2PA responses close to 800 nm than dye D1 whereas dye D1 shows larger 2PA responses at 950 nm and in the 1050–1100 nm region.

### Preparation and characterisation of nanoemulsions loaded with dyes D1 and D2

NEs loaded with the D1 and D2 dyes were then prepared according to a previously reported procedure.^31^ Briefly, the dye is dissolved in an oily phase containing Lipoid® E80 (a mixture of egg yolk lecithin designed for parenteral use) in Miglyol® 840. An aqueous phase containing Tween® 80 (a polysorbate emulsifier used in parenteral formulation) is added and the NE formulations are obtained after emulsification and homogenisation by sonication. Turbid orange NE[D1] and purple NE[D2] emulsions were realized.

**Fig. 4:**
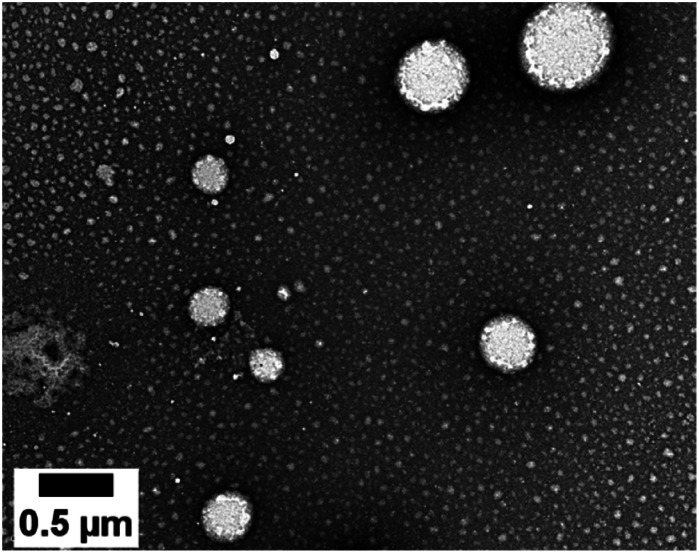
TEM image of the NEs loaded with the D2 dye.

The dye loading did not impair the NE diameter or polydispersity index (PdI) (Tables 2 and S1†). The TEM image (Fig. 4) shows that the nanodroplets have a spherical shape with a size ranging from 60 to 600 nm in line with the Gaussian distribution obtained by DLS.

**Table 2:** Properties of the dye-loaded NEs

Dye	*∅* a, nm	Zeta potential, mV	*λ* ^abs^_max_, nm	*λ* ^em^_max_, nm	*Φ*_f_	*N* b	*λ* ^2PA^_max_, nm	*Nσ* ^max^_2_ *Φ*_f_ c, GM
D1	143	16.2	495	707	0.05^d^	3500	1050	10^5^
							1020	7.7 × 10^4^
							940	6.4 × 10^4^
							820	4.2 × 10^4^
D2	151	15.9	540	742	0.06^d^	2080	1080	1.4 × 10^5^
							960	5 × 10^4^
							<830	>7.1 × 10^4^

a Droplet average diameter determined by DLS.

b Average number of D1 or D2 dye molecules per droplet.

c NE droplet’s two-photon brightness.

d Standard: DCM in EtOH (*Φ*_f_ = 0.437).

A colloidal stability study of the NEs was performed over a period of one year by evaluating the evolution of the droplet diameter and PdI (Table S2†). A slight increase of the PdI and mean diameter was noticed over time, but the formulation diameter remained submicronic and monodisperse (PdI < 0.2).^32^

Quite interestingly, the dye loading inside NEs was found to induce a change (reversal) in zeta potential. Compared to control NEs (*ζ* = −32 mV), the NE[D1] and NE[D2]s show a positive zeta potential (*ζ* = 16.2 mV and 15.9 mV). This finding reveals that the encapsulation of the D1 and D2 dyes modifies the surface properties of the NEs. This positive potential is consistent with dyes D1 and D2 incorporated in the lipid corona of the NEs with their positive end-group pointing to the aqueous phase and located at the interface (Scheme 2). The slightly lower zeta potential of NE[D2] than that of NE[D1] is consistent with the less marked positive charge on the quinolinium end-group due to the different electronic distribution in the ground state of dye D2.

### Linear and nonlinear optical properties of the NEs

**Fig. 5:**
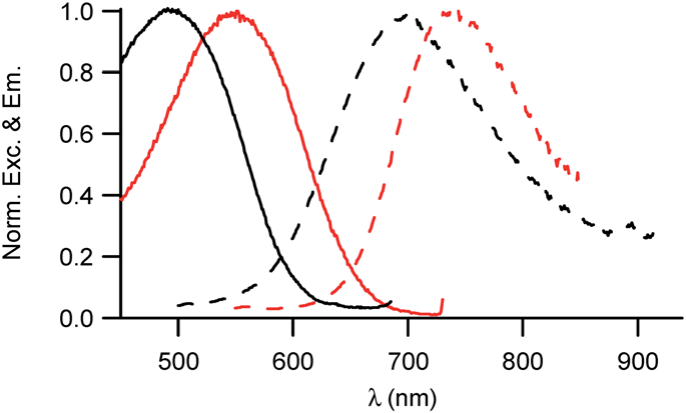
Excitation and emission spectra of NE[D1] (black) and NE[D2] (red).

The investigation of the optical properties of NE[D2] further confirms the positioning of the D1 and D2 dyes within NEs and provides additional information on their orientation (Fig. 5).

We note that the excitation spectra of the dyes when encapsulated in NE emulsions are significantly blue-shifted (by 1250 cm^−1^ for both dye D1 and D2) in comparison with the absorption spectra of the dyes in toluene (Tables 1 and 2). This blue-shift reveals a destabilisation of the Franck–Condon excited state. This is consistent with a photo-induced intramolecular charge transfer from the donor end-group to the acceptor moiety leading to a fractional positive charge on the donor end-group in the excited state (Fig. 1) and a decrease of the positive charge on the acceptor end-group. Due to the positioning of the donor end-group in the oily core (where no negative counter-ion is present), and the lower positive charge at the water interface, the FC excited state is thus destabilized. In contrast, we note that the emission characteristics are similar to those measured in toluene (Tables 1 and 2). This suggests that the dyes tend to adopt a more tilted orientation in the relaxed emitting state. As a result, both dye loaded NEs show NIRF emission (Fig. 5). The fluorescence of NE[D2] remains red-shifted compared to that of NE[D1], as is the case for its absorption band.

In order to evaluate the brightness of the NE droplets, the number of dye molecules incorporated in each droplet was determined. The droplet concentration was measured using nanoparticle tracking analysis. The dye concentration was measured by absorption after lyophilisation of a NE aliquot and subsequent dissolution in chloroform of the dyes which are well soluble as isolated units in that solvent. This protocol allows us to get rid of scattering and to rule out any shadowing or aggregation effect which may occur in NEs. Thanks to their amphiphilic character, high loading of dyes could be achieved, with loading amounting to 2000 to 3500 dye molecules per nanodroplet (Table 2). Due to the encapsulation of a large number of strongly absorbing dye molecules per droplet, the NEs were thus found to show very large brightness as NIRF contrast agents (respectively 7 × 10^6^ M^−1^ cm^−1^ and 5 × 10^6^ M^−1^ cm^−1^ for dye D1 and D2), demonstrating their potential as contrast agents for NIRF *in vivo* imaging.

**Fig. 6:**
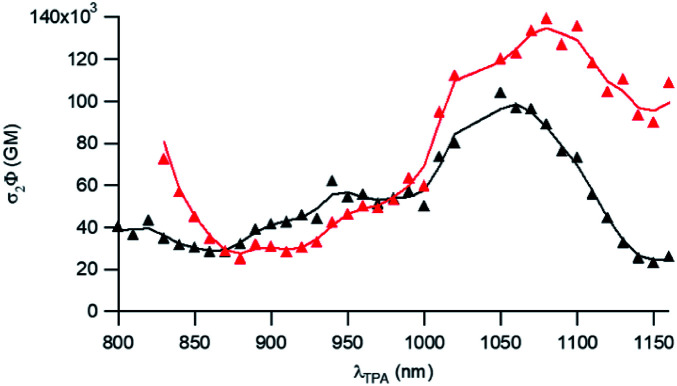
Two-photon brightness of NE[D1] (black) and NE[D2] (red).

To further attest the potential of NE[D1] and NE[D2] for *in vitro* multiphotonic bioimaging, we investigated the two-photon absorption properties of these nano-objects by using two-photon excited fluorescence (TPEF) spectroscopy. As illustrated in Fig. 6, both NEs show very large two-photon absorption with the lowest energy 2PA maximum located at 1050 and 1080 nm, respectively, for NE[D1] and NE[D2] with a very large 2PA response in the 1000–1100 nm range. A second local maximum is observed around 950 nm.

As a result, the nanodroplets show very high two-photon brightness (Table 2), making them of high interest as fluorescent probes for NIR (two-photon excitation) to NIR (NIRF detection) *in vitro* imaging. In particular, both NE[D1] and NE[D2] show two-photon brightness of about 10^5^ GM in the 1020–1050 nm region, with NE[D2] showing somewhat larger brightness (Fig. 6). On the other hand, NE[D2] shows larger two-photon brightness than NE[D1] close to 800 nm. Finally, NE[D1] and NE[D2] show similar two-photon brightness (*i.e.* 5 × 10^4^ GM) around 950 nm. Hence these three wavelengths will be preferred for monitoring cellular uptake of the NEs by two-photon fluorescence imaging.

### Investigation of dye loaded NE internalisation in cancer cells by multimodal optical imaging

**Fig. 7:**
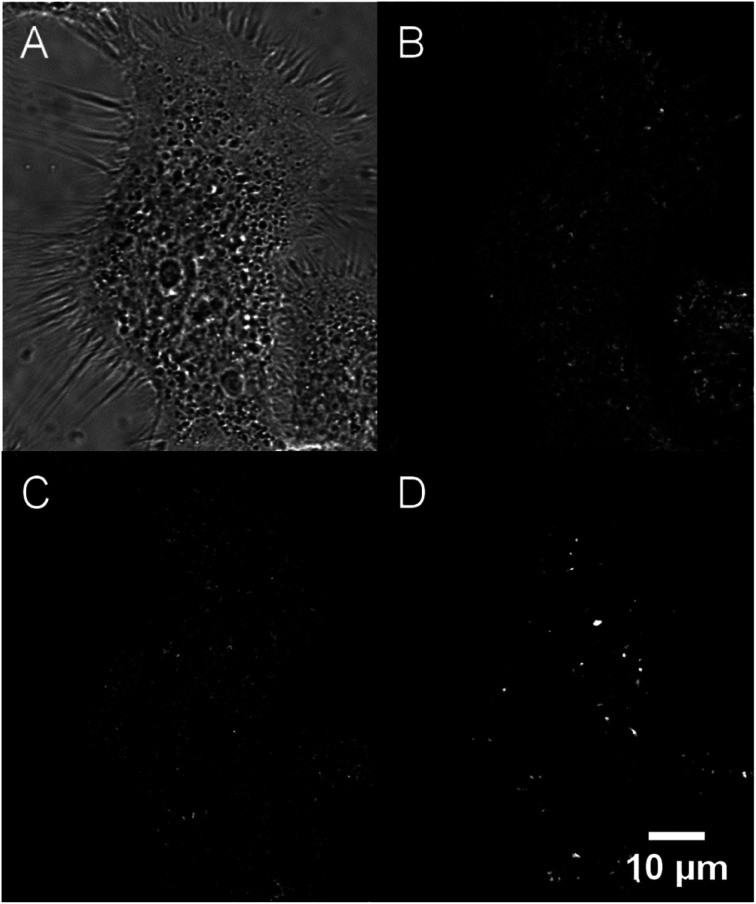
Confocal imaging of live A431 cells incubated with NE[D2]. (A) Bright-field. (B) Fluorescence imaging (*λ*^exc^ = 561 nm). (C) Two-photon imaging (*λ*^exc^ = 950 nm). (D) Second Harmonic Generation (SHG) imaging (*λ*^exc^ = 896 nm). Except for bright-field and SHG, photons were collected in the 650–800 nm range.

Their large (one- and two-photon) brightness values make NE[D1]s and NE[D2]s suitable for monitoring the fate of dye-loaded NEs in cellular environments and the kinetics of their cell internalisation. Having in mind their potential use as theranostic tools, we first investigated their internalisation into cancer cells. We previously checked the safety of the NE[D1], NE[D2] as well as the NE[D2]-PEG and NE[D2]-PEG nanocarriers (Fig. S5†). The ability of the bare NE[D2] nanocarriers was estimated in epidermoid carcinoma A431 cancer cells. Cellular uptake of the NIRF-NEs was confirmed by both confocal and two-photon fluorescence microscopy on living cells. As shown in Fig. 7, an intense fluorescence signal is detected in the whole cytoplasm but none in the nucleus (Fig. 7A and B). The NIR fluorescence of the dye and its large brightness allowed reduction of autofluorescence thus revealing a significant uptake of the D2 dye into the cells (Fig. 7A and B). Moreover, the optical properties of the dye allow both 2P excitation (950 nm) and detection (650–800 nm range) in the NIR range, resulting in total suppression of background fluorescence (Fig. 7C) and revealing the precise localisation of the dye in the cellular environment. In particular, we observe that numerous “hot spots” concentrate a large amount of the fluorescence signals suggesting accumulation of the dye molecules in specific subcellular compartments. Their tiny size suggests that they are endosomes which have trapped the NEs during the endocytosis process. Interestingly, Second Harmonic Generation (SHG) imaging provides additional information. As noted from Fig. 7D, these spots generate SHG signal, indicating that the dye D2 is oriented in an asymmetric way. This reveals that D2 dye molecules are located in the outer leaflet of the lipid membrane as a result of fusion between the NEs and the membrane of the endocytosis vesicles. The push–pull nature of the dye combined with its confinement and preferential orientation into the vesicle's membrane makes it a powerful SHG emitter^27–29^ explaining the strong SHG signal which exclusively arises from the vesicles.

In order to use these dye-loaded NEs for *in vivo* imaging of tumours, stealth strategies were developed to reduce opsonisation with plasma proteins and to prolong the circulation time of the engineered NIRF-NEs. The gold standard strategy is to create a dense layer of polyethylene glycol (PEG) macromolecules on the nanodroplet surface. PEGylation reduces non-specific protein adsorption and cellular uptake.^33^ PEG has the advantages of low-toxicity and low-immunogenicity. Based on this, we prepared PEGylated NEs loaded with dye D1 and D2. Their optical properties were found to be similar to those of bare NEs (Table S3†). We note that the dye-loaded PEGylated NEs show negative surface potentials due both to the negative charge of the DSPE PEGylated lipid and negatively charged phospholipids (*i.e.* phosphatidylethanolamine) contained in lecithin used for the preparation of stealth nanoemulsions (see the Experimental section).

**Fig. 8:**
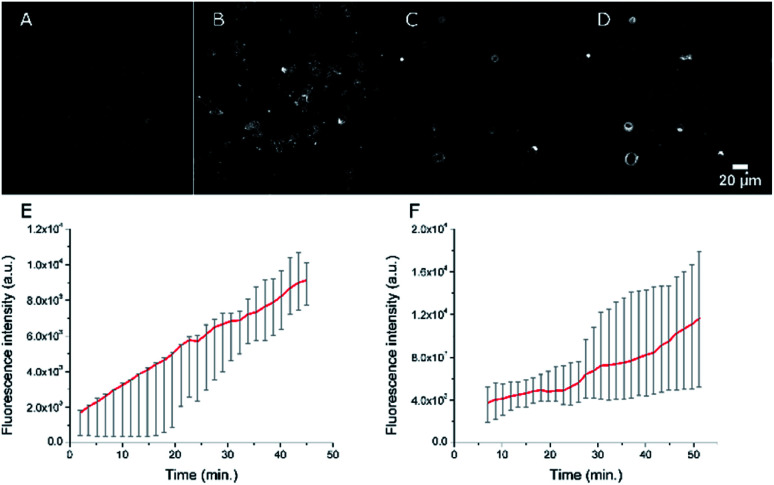
Confocal imaging of the accumulation of NEs in live U87 cells. Fluorescence imaging (*λ*^exc^ = 561 nm) 2 min (A) and 42 min (B) after the addition of NE[D1]-PEG. Fluorescence imaging (*λ*^exc^ = 561 nm) 7 min (C) and 47 min (D) after the addition of NE[D2]-PEG. (E) Averaged quantification of the fluorescence signal of NE[D1]-PEG (red line) in U87 cells (grey error bars, *n* = 5). (F) Averaged quantification of the fluorescence signal of NE[D2]-PEG (red line) in U87 cells (grey error bars, *n* = 5). The photons were collected in the 610–800 nm range. Changes of the averaged fluorescence signal with time were calculated on 5 cells and for both NEs a linear increase of accumulation of the fluorescence signal in the cells is seen.

As our final goal was to develop nano-tools as theranostics agents and adjuvant treatment during surgery of glioblastoma cancer, we first performed multimodal optical imaging on U87 cells which are model cancer cells for glioblastoma. These studies were aimed at gaining information on the kinetics of the cell internalisation of the dye-loaded NEs into the cancer cells. Our experiments do show that the uptake of the PEGylated NEs in U87 cells is indeed fast. As soon as the U87 cells were incubated with NEs, we could observe the uptake of the NEs in the first few minutes (Fig. 8, Movies S1 and S2†). The monotonic increase of the fluorescence signal with time suggests an enhancement of the concentration of the NEs in cells (five-fold and three-fold increase for the NE[D1]-PEG and NE[D2]-PEG, respectively) during the acquisition time (43 min). Hence NE[D1]-PEG is internalised more rapidly than NE[D2]-PEG.

**Fig. 9:**
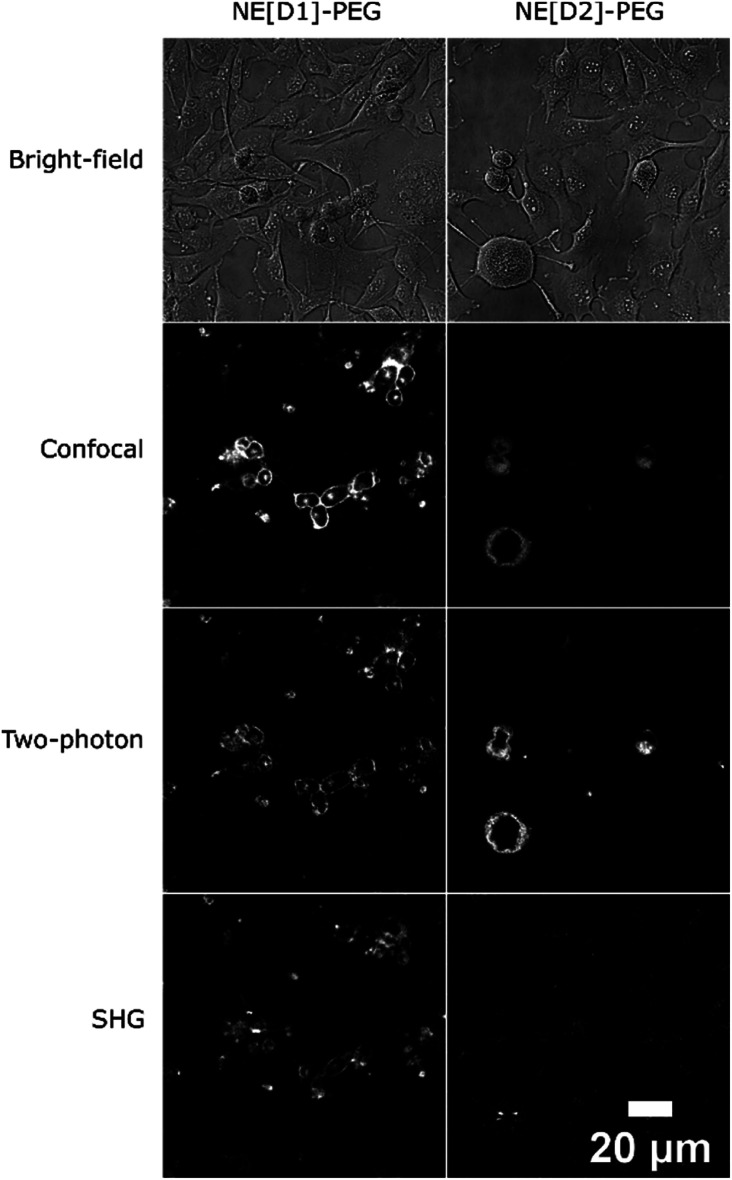
Multimodal optical imaging of NEs in live U87 cells. Bright-field, confocal imaging (*λ*^exc^ = 561 nm), two-photon imaging (*λ*^exc^ = 1020 nm for NE[D1]-PEG, *λ*^exc^ = 820 nm for NE[D2]-PEG) and second harmonic generation imaging (*λ*^exc^ = 896 nm) of live U87 cells incubated with NEs. Except for bright-field and SHG, photons were collected in the 610–800 nm range.

The fate of the NEs formulated with DSPE-PEG can be monitored by two-photon (Fig. S7†) and SHG microscopy as well (Fig. 9). Strikingly, the SHG imaging suggests that the dye D1 is localized both in the outer cell membrane (indicating fusion of the NEs with the cell membrane) and endocytosis vesicles, while the SHG response generated by dye D2 is lower and located only in the endosomes. The lower SHG signal generated by dye D2 might be related to a less favourable orientation (tilt angle as well as orientational disorder)^26,30^ in the lipid membrane due to its different electronic distribution and shorter size than that of dye D1. Indeed the more strongly electron-withdrawing hydrophilic moiety and the shorter conjugated connector of the push–pull dye D2 induce a more pronounced partial intramolecular charge transfer in the ground state.^34^ This results in an electronic distribution closer to the so-called cyanine limit for dye D2. Such a difference is confirmed by the red-shifted absorption and a smaller Stokes shift value of dye D2 than that of dye D1 (Table 1). This different electronic distribution results in a less marked positive charge on the hydrophilic end-group of D2 dye and concomitant slight positive charge on the donating hydrophobic end-group. Such a variation is less favourable to the ideal orientation shown in Scheme 2. We note that a tilted orientation was reported for an amphiphilic push–pull membrane probe having the same hydrophilic moiety.^26^ The resonance factor is also expected to significantly increase the SHG action cross-section (*σ*_SHG_) of dye D1 compared to that of dye D2. Yet, increasing the incubation time of NE[D2]-PEG to two hours leads to an enhancement of the SHG response (Fig. S6†). This further confirms its presence in endocytosis vesicles and slower internalisation.

**Fig. 10:**
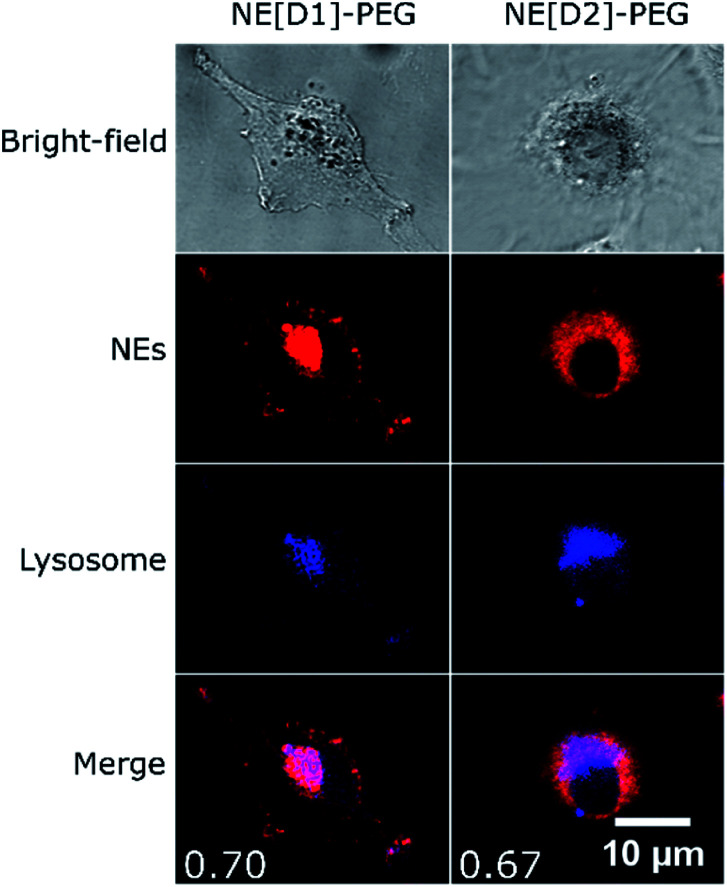
Co-localization imaging of NEs with lysosomes in live U87 cells. Bright-field, confocal imaging of NEs (*λ*^exc^ = 561 nm), confocal imaging of LysoTracker Blue DND-22 (*λ*^exc^ = 405 nm) and the merged image of NEs and LysoTracker confocal imaging of live U87 cells incubated with NE[D1]-PEG or NE[D2]-PEG and LysoTracker Blue DND-22. Values of Pearson's correlation coefficient are indicated as insets in the merged images. The photons were collected in the 610–800 nm range for NEs and the 415–480 nm range for LysoTracker.

Co-localization experiments were then conducted to gain information on the fate of the dyes after internalisation of the NEs by endocytosis. The co-localization experiments with LysoTracker (Fig. 10, S8 and S9†) clearly evidence the presence of the dyes in the lysosomes logically resulting from the fusion of endocytosis vesicles with lysosomes.

**Fig. 11:**
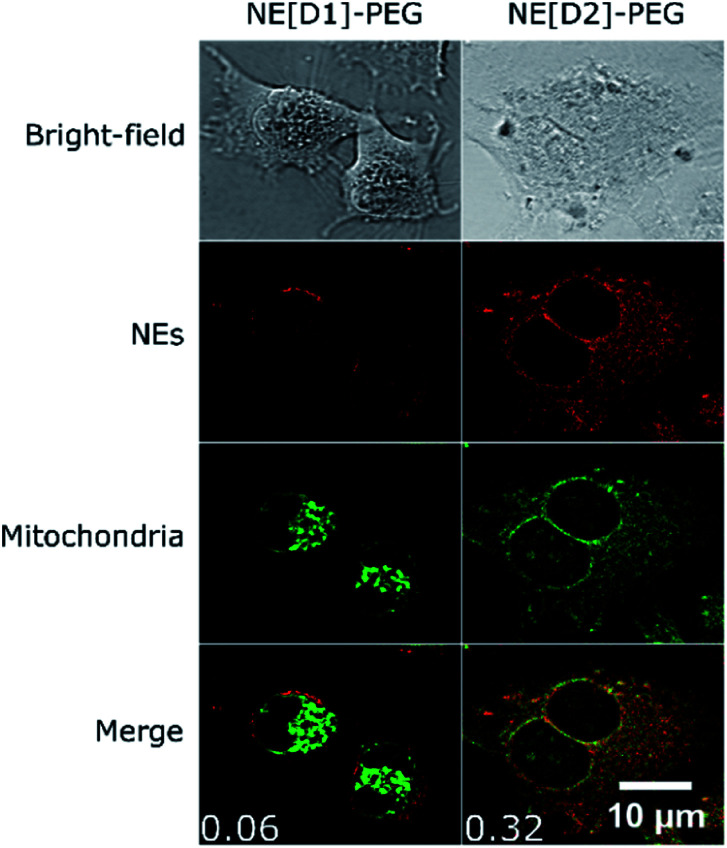
Co-localization imaging of NEs with mitochondria in live U87 cells. Bright-field, confocal imaging of NEs (*λ*^exc^ = 561 nm), confocal imaging of MitoTracker GreenFM (*λ*^exc^ = 488 nm) and the merged image of NEs and MitoTracker confocal imaging of live U87 cells incubated with NE[D1]-PEG or NE[D2]-PEG and MitoTracker Green FM. Values of Pearson's correlation coefficient are indicated as insets in the merged images. The photons were collected in the 610–800 nm range for NEs and the 500–550 nm range for MitoTracker.

Interestingly, we also observed co-localization of the fluorescence in the mitochondria in the case of U87 cells incubated with NE[D2]-PEG. Such a phenomenon was not observed upon incubation with NE[D1]-PEG (Fig. 11, S10 and S11†). We also note that U87 cell membranes are stained only when incubated with NE[D1]-PEG (and not with NE[D2]-PEG) which is in good agreement with SHG imaging data. This reveals that the nature of the dye modifies the internalisation pathways. NE-loading with dye D2 allows NE[D2]-PEG to partly escape from the lysosomes to reach other organelles and particularly mitochondria, whereas fusion of NE[D1]PEG with the U87 cell membrane occurs.

### *In vivo* NIRF imaging with stealth NEs tagged with dyes D1 and D2

**Fig. 12:**
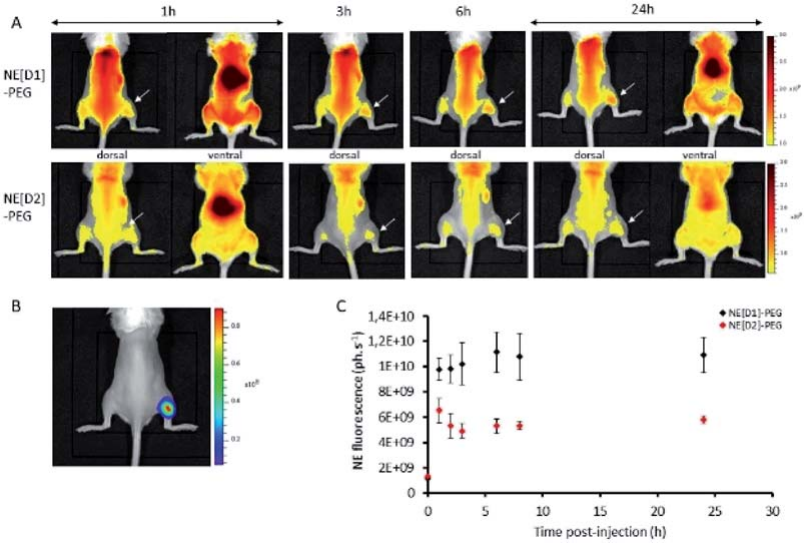
Time course of D1 and D2 fluorescence signal in mice bearing subcutaneous tumours. (A) Fluorescence reflectance imaging of a representative mouse at different times after NE[D1]-PEG or NE[D2]-PEG injection. (B) Bioluminescence imaging of a representative mouse. (C) Quantification of fluorescence signal in the tumour after NE[D1]-PEG (black) or NE[D2]-PEG (red) injection (mean ± standard deviation for *n* = 3).

*In vivo* experiments were performed using immunocompetent black-6 albino mice bearing a bioluminescent subcutaneous tumour. Murine prostate cancer cells (RM1-CMV-lucF) were injected subcutaneously in the posterior right leg of mice. Four days after injection of cells, tumour growth was monitored by bioluminescence imaging (BLI) (Fig. 12B). NE[D1]-PEG and NE[D2]-PEG formulations were first spotted on glass slides to ensure a similar fluorescent signal and then NE[D1]-PEG and NE[D2]-PEG (20 μL diluted in 180 μL of 5% glucose) were administered intravenously *via* the tail vein, and their distribution was monitored by fluorescence reflectance imaging (FRI) over 24 hours after injection. As shown in Fig. 12A, the fluorescent signal from NE[D1]-PEG and NE[D2]-PEG was first dispersed by the blood flow in the mouse tissues but an increase of the fluorescent signal was detected in the liver as soon as 1 hour after NE injections. It should be noted that due to photon absorption by tissues, the fluorescent signal in the liver (deep organ) is underestimated in comparison to the superficial tissues. Both the whole body labelling and the liver labelling fade over time. Part of the fluorescence signal is also detected within the tumours. We note that the fluorescence level in NE[D1]-PEG injected mice is higher than in NE[D2]-PEG injected mice both in liver and tumours. Quantification of the fluorescence signals in the tumours further confirmed this observation (Fig. 12C). However, the fluorescence signal in tumours remained low when compared to the fluorescent signal in the liver on excited organs 24 hours after injection (Fig. S12†).

The *in vivo* NIRF fluorescence pattern is consistent with previously reported data using the RM1 sub-cutaneous mouse model after injecting NEs^15^ or other types of nanoparticles.^35^ This is also consistent with many other types of nanoparticles and tumour models in mice. Accumulation in the liver results from fluorescent probe capture by Kupffer cells while accumulation in the tumour originates from the enhanced permeability and retention (EPR) effect.^36^ The much lower fluorescence signal observed after injection with NE[D2]-PEG cannot be related to the excitation (535 nm) or imaging conditions (detection with the Cy5.5 filter in the 695–770 nm range) as they are more favourable to NE-[D2]-PEG than to NE-[D1]-PEG (see Fig. 5). This suggests that the NE metabolism and carrier behaviour are influenced by the presence of the dyes which modify their *in vivo* properties. This parallels the *in vitro* observation of slower kinetics of internalisation of NE[D2]-PEG in U87 cells than those of NE[D1]-PEG.

## Conclusions

Tailor-made amphiphilic elongated dyes having thiophene units in their conjugation path have been synthesized. They combine NIR emission in the 600–800 nm range, sizeable Stokes shift values, large two-photon absorption in the whole 800–1100 nm range (thus allowing NIR to NIR excitation/emission by two-photon excitation) and large second-order NLO response. These dyes were demonstrated to show high loading in NEs which are attractive nanocarriers for pharmaceutical use. A few thousand molecules of these dyes could be loaded in the lipid corona of NEs. As a result NIR emitting NEs showing very high one- and two-photon brightness were obtained. Strikingly the incorporation of these dyes was also shown to affect the properties of the NEs, conferring positive surface potential to NEs.

Thanks to the unique linear and nonlinear optical properties of the dyes, the carrier behaviour of the dye-loaded NEs into live cancer cells could be monitored by multimodal nonlinear optical imaging (*i.e.* combined TPEF and SHG microscopies) clearly evidencing the endocytosis process *via* SHG monitoring of endocytosis vesicles. Interestingly, the kinetics of the internalisation of the PEGylated dye-loaded NEs into live U87 cancer cells (model cells for glioblastoma) was found to depend on the electronic distribution of the dye. The hemicyanine dye having the positive charge more confined on the acceptor hydrophilic end-group shows the fastest internalisation, intermediate accumulation in the cell membrane and subsequent uptake in lysosomes. In contrast, the dye having a more delocalised distribution of positive charge (*i.e.* closer to the cyanine type) shows slower internalisation into cancer cells as well as partial uptake in the mitochondria and no accumulation in cell membranes. Interestingly, the NEs showing the fastest internalisation into U87 cancer cells were also shown to allow *in vivo* NIRF imaging of tumours in mice. As such the multimodal NEs are of much interest and could be further elaborated for the design of theranostic multifunctional nanoparticles by loading lipophilic cytotoxic drugs in the oily core and grafting targeting agents (such as fragment of antibodies^37^) on the surface to yield new theranostics agents and adjuvant treatment during surgical resection of tumours.

## Experimental

### Materials and methods

All air- or water-sensitive reactions were carried out under an argon atmosphere. Solvents were distilled freshly using standard protocols. The starting materials were purchased from Aldrich, TCI and Alfa Aesar. Reactions were monitored by thin layer chromatography on Merck silica gel 60F254 precoated aluminium sheets. Column chromatography was performed on Fluka silica gel 60 (40–63 μm). ^1^H, ^31^P and ^13^C NMR spectra were recorded on Bruker Avance I 300, Avance II 400, and Avance III 600 spectrometers. Chemical shifts are given in parts per million with respect to the residual solvent peak, and coupling constants (*J*) are given in Hertz. Melting points were measured on a Stuart SMP 10. IR spectra were recorded on a PerkinElmer Spectrum 100 FT-IR spectrometer on solid samples. High-resolution mass spectroscopy was performed at the CESAMO (Bordeaux, France) using an FD emitter. LC/MS analyses were performed on a Shimadzu LCMS-2020. Elemental analyses were carried out at the ‘‘Institut de Chimie des Substances Naturelles’’ (Gif-sur-Yvette, France). Pharmaceutical neutral oil phase Miglyol® 840 (oil based on propylene glycol diester of C8 and C10 saturated plant fatty acids) was provided by Cremer Oleo GmbH & Co. KG (Hamburg, Germany). FDA approved surfactants were acquired from the following companies: egg lecithin containing 82.3% phosphatidylcholine (Lipoid® E80, Lipoid GmbH Ludwigshafen, Germany); Polysorbate 80 (Tween® 80, SEPPIC, France). PEGylated phospholipids PE 18:0/18:0-PEG 2000 (*N*-(carbonyl-methoxypolyethylenglycol-2000)-1,2-distearoyl-*sn-glycero*-3-phosphoethanolamine) were kindly provided by Lipoid. Glycerol was purchased from Cooperation Pharmaceutique Française (Melun, France). Glucose 5 (G5) was acquired from Baxter (Guyancourt, France).

### Synthetic procedures

#### Synthesis of the dyes D1 and D2

##### 5′-(4-(dibutylaminophenyl)bis-2,2′-thiophene)carboxaldehyde (2b)

To an oven dried round bottom flask cooled under argon were added 4-iodo-*N*,*N*-dibutylaniline (1.21 g, 3.65 mmol), 5′-formyl-2,2′-bithiophene-5-boronic acid (0.95 g, 4.01 mmol), K_2_CO_3_ (1.25 g, 9.12 mmol), Pd(dppf)Cl_2_ (148 mg, 0.18 mmol), toluene (9 mL) and methanol (9 mL). The mixture was stirred at 80 °C for 6 h. The mixture was filtered through a pad of Celite, rinsed with dichloromethane and then the filtrate was concentrated under vacuum. The residue was further purified by silica-gel column chromatography using a 1/1 dichloromethane/petroleum ether mixture to yield 1 g (73%) of the desired compound 2b as an orange solid. Mp = 110–111 °C. ^1^H NMR (300 MHz, CDCl_3_) *δ* 9.83 (s, 1H), 7.65 (d, *J* = 4 Hz, 1H), 7.45 (d, *J* = 8.9 Hz, 2H), 7.29 (d, *J* = 3.8 Hz, 1H), 7.20 (d, *J* = 4 Hz, 1H), 7.07 (d, *J* = 3.9 Hz, 1H), 6.63 (d, *J* = 8.9 Hz, 2H), 3.30 (t, *J* = 7.6 Hz, 4H), 1.59 (quint, *J* = 7.5 Hz, 4H), 1.37 (sext, *J* = 7.4 Hz, 4H), 0.96 (t, *J* = 7.3 Hz, 6H); ^13^C {^1^H} NMR (75 MHz, CDCl_3_) *δ* 182.2, 148.1, 148.0, 147.8, 140.6, 137.5, 132.2, 127.3, 126.9, 123.1, 121.3, 120.3, 111.5, 50.7, 29.4, 20.3, 13.9. IR (KBr): 2955, 1661, 1606, 1456, 1231, 1052, 791 cm^−1^. HRMS (*m*/*z*) [M] for C_23_H_27_NOS_2_ calcd = 397.15341; found = 397.15423. Elemental analysis for C_23_H_27_NOS% calcd: C, 69.48; H, 6.84; N, 3.52; found: C, 69.35; H, 7.00; N, 3.56.

##### 4-(Diphenylphosphinoylmethyl)pyridine (3p)

4-Picoline (3.07 g, 33 mmol, 3.2 mL) was dissolved in 100 mL of anhydrous THF. The solution was cooled to −78 °C and 1 equivalent of LDA (obtained *in situ* with 33 mmol, 14 mL of 2.36 M BuLi in hexane added to 33 mmol, 3.31 g (4.5 mL) of anhydrous diisopropylamine in 23 mL of anhydrous THF) was added dropwise. After leaving the orange solution at −78 °C for 2 h, chlorodiphenylphosphine (7.2 g, 33 mmol, 5.9 mL) was added and the solution was stirred for 30 minutes at −78 °C. Then, the reaction mixture was slowly allowed to warm to room temperature. The solvent was concentrated under vacuum, and the residue was extracted with dichloromethane and the organic phase was washed with water, and dried over anhydrous Na_2_SO_4_. The solvent was concentrated under vacuum. The obtained oily product was dissolved in 150 mL of toluene and the solution was refluxed while bubbling with air for 16 h. After cooling to room temperature, the mixture was filtered and the solid was collected and rinsed with toluene to afford 2.95 g of 3p as a white solid (yield 33%). ^1^H NMR (CDCl_3_, 300 MHz): *δ* 8.40 (d, *J* = 5.9 Hz, 1H), 7.66–7.73 (m, 4H), 7.43–7.54 (m, 6H), 7.48 (d, *J* = 8.1 Hz, 2H), 7.03–7.06 (m, 2H), 3.62 (d, *J* = 13.9 Hz, 2H); ^31^P (CDCl_3_, 121.4 MHz): *δ* 28.6.

##### *N*,*N*-Dibutyl-4-((5-(quinolin-4-yl)thiophen-2-yl)ethynyl)aniline (4a)

3q (306 mg, 1.04 mmol), 2a (300 mg, 0.95 mmol), NaH (60% in mineral oil, 46 mg, 1.90 mmol) and 18-C-6 crown ether (catalytic amount) were suspended in 20 mL of THF. The mixture was stirred at room temperature overnight. The excess of NaH was quenched by slow addition of a small amount of water to the reaction mixture and then THF was evaporated under vacuum. The product was extracted with dichloromethane, and the organic phase was washed with water, dried over anhydrous Na_2_SO_4_, filtered, and concentrated under reduced pressure. The residue was purified by silica-gel column chromatography using an 8/2 dichloromethane/ethyl acetate mixture to yield 350 mg (85%) of 4a as a red oil. ^1^H NMR revealed the presence of a small proportion of the *Z* isomer (less than 10%). ^1^H NMR (600 MHz, CDCl_3_) *E* isomer: *δ* 8.86 (d, *J* = 4.6 Hz, 1H), 8.23 (d, *J* = 8.4 Hz, 1H), 8.11 (d, *J* = 8.4 Hz, 1H), 7.73 (t, *J* = 7.5 Hz, 1H), 7.59 (t, *J* = 7.5 Hz, 1H), 7.56 (d, *J* = 4.6 Hz, 1H), 7.54 (d, *J* = 15.8 Hz, 1H), 7.50 (d, *J* = 8.7 Hz, 2H), 7.46 (d, *J* = 15.7 Hz, 1H), 7.12 (d, *J* = 3.6 Hz, 1H), 7.08 (d, *J* = 3.6 Hz, 1H), 6.65 (d, *J* = 8.7 Hz, 2H), 3.31 (t, *J* = 7.5 Hz, 4H), 1.60 (quint, *J* = 7.6 Hz, 4H), 1.38 (sext, *J* = 7.4 Hz, 4H), 0.97 (t, *J* = 7.3 Hz, 6H). ^13^C {^1^H} NMR (75 MHz, CDCl_3_): *δ* 150.0, 148.7, 148.0, 146.6, 142.6, 138.7, 130.0, 129.8, 129.2, 128.2, 126.9, 126.3, 126.1, 123.4, 123.1, 120.9, 120.0, 116.1, 111.6, 50.7, 29.4, 20.3, 14.0. IR (KBr): 1605, 1574, 1514, 1463, 1451, 1367, 1301, 1224, 1191, 1055, 944, 812, 756 cm^−1^. MS [M + H^+^]: 441.05. Elemental analysis calculated for C_29_H_32_N_2_S%: C, 79.05; H, 7.32; N, 6.36; found: C, 78.67; H, 7.40; N, 6.28.

##### *N*,*N*-Dibutyl-4-((5-(pyridin-4-yl)bis-2,2′-thiophen-5′-yl)ethynyl)aniline (4b)

3p (280 mg, 0.86 mmol), 2b (361 mg, 0.91 mmol), NaH (60% in mineral oil, 44 mg, 1.80 mmol) and 18-C-6 crown ether (catalytic amount) were suspended in 20 mL of THF. The mixture was stirred at room temperature overnight. The excess of NaH was quenched by slow addition of a small amount of water to the reaction mixture and then the THF was concentrated under vacuum. The obtained precipitate was collected by filtration, and washed with diethyl ether to afford the product as an orange solid (330 mg, 81%). Mp = 190 °C. ^1^H NMR (300 MHz, CDCl_3_) *δ* 8.54 (d, *J* = 6.1 Hz, 2H), 7.44 (d, *J* = 8.8 Hz, 2H), 7.37 (d, *J* = 15.9 Hz, 1H), 7.31 (d, *J* = 6.1 Hz, 2H), 7.14 (d, *J* = 3.8 Hz, 1H), 7.05 (m, 3H), 6.75 (d, *J* = 16.0 Hz, 1H), 6.63 (d, *J* = 8.8 Hz, 2H), 3.30 (t, *J* = 7.6 Hz, 4H), 1.59 (quint, *J* = 7.6 Hz, 4H), 1.37 (sext, *J* = 7.4 Hz, 4H), 0.96 (t, *J* = 7.3 Hz, 6H). ^13^C {^1^H} NMR (75 MHz, CDCl_3_): *δ* 150.1, 147.8, 145.4, 144.3, 139.5, 138.6, 133.5, 129.2, 126.8, 125.9, 125.1, 124.6, 123.2, 121.1, 120.8, 120.4, 111.6, 50.7, 29.4, 20.3, 13.9. IR (KBr): 1606, 1591, 1504, 1454, 1384, 1192, 804, 788 cm^−1^. HRMS (*m*/*z*) [M] for C_29_H_32_N_2_S_2_ calcd = 472.20069; found = 472.20221. Elemental analysis for C_29_H_32_N_2_S_2_% calcd: C, 73.68; H, 6.82; N, 5.93; found: C, 73.21; H, 6.46; N, 5.82.

##### D1

To a solution of 4b (60 mg, 0.12 mmol) in 1.5 mL of chloroform were added 23 μL of dimethyl sulfate (32 mg, 0.25 mmol) under argon and the reaction mixture was stirred at room temperature overnight. Then, the solvent was partially evaporated and diethyl ether was added. The obtained precipitate was filtered, rinsed with diethyl ether and dried under vacuum to give 64 mg (90%) of compound D1 as a black solid. Mp = 176 °C. ^1^H NMR (300 MHz, CDCl_3_) *δ* 8.62 (d, *J* = 6.7 Hz, 2H), 7.66 (d, *J* = 6.7 Hz, 2H), 7.68 (d, *J* = 15.5 Hz, 1H), 7.37 (d, *J* = 8.7 Hz, 2H), 7.23 (d, *J* = 3.9 Hz, 1H), 7.07 (d, *J* = 3.8 Hz, 1H), 7.06 (br, 2H), 6.52–6.59 (m, 1H_8_, 2H), 4.28 (s, 3H) 3.78 (s, 3H). 3.27 (br, 4H), 1.53–1.63 (quint, *J* = 7.3 Hz, 4H), 1.35 (sext, *J* = 7.4 Hz, 4H), 0.96 (t, *J* = 7.3 Hz, 6H). ^13^C {^1^H} NMR (50 MHz, CDCl_3_): *δ* 152.6, 148.0, 146.6, 144.4, 142.9, 137.8, 134.6, 134.4, 132.6, 126.8, 126.4, 123.7, 123.0, 121.3, 120.3, 119.4, 111.5, 54.5, 50.7, 47.2, 29.3, 20.3, 13.9. IR (KBr): 1641, 1604, 1518, 1453, 1252, 1216, 1187, 1052, 1012, 806, 745 cm^−1^. *m*/*z* calcd for [C_30_H_35_N_2_S_2_]^+^ MS (M)^+^: 486.95; found: 486.95.

##### D2

To a solution of 4a (54 mg, 0.12 mmol) in 3.5 mL of diethyl ether were added 25 μL of dimethyl sulfate (34 mg, 0.27 mmol) under argon and the reaction mixture was stirred at room temperature overnight. The obtained precipitate was filtered, rinsed with diethyl ether and dried under vacuum to give 60 mg (86%) of the dye as a black solid (mp = 207 °C). ^1^H NMR (300 MHz, CDCl_3_) *δ* 9.31 (d, *J* = 6.6 Hz, 1H), 8.41 (d, *J* = 8.5 Hz, 1H), 8.07–8.12 (m, 2H), 8.02 (t, *J* = 7.5 Hz, 1H), 7.91 (d, *J* = 15.3 Hz, 1H), 7.78–7.84 (t, *J* = 7.5 Hz, 1H), 7.44 (d, *J* = 8.5 Hz, 2H), 7.34 (d, *J* = 3.9 Hz, 1H), 7.28 (d, *J* = 15.3 Hz, 1H), 7.07 (d, *J* = 3.9 Hz, 1H), 6.62 (d, *J* = 8.5 Hz, 2H), 4.49 (s, 3H) 3.80 (s, 3H). 3.32 (t, *J* = 7.4 Hz, 4H), 1.60 (quint, *J* = 7.3 Hz, 4H), 1.38 (sext, *J* = 7.4 Hz, 4H), 0.97 (t, *J* = 7.3 Hz, 6H). ^13^C {^1^H} NMR (75 MHz, CDCl_3_): *δ* 152.4, 152.1, 148.8, 148.0, 138.7, 137.2, 136.8, 136.2, 134.7, 128.8, 127.3, 125.9, 125.4, 121.9, 119.8, 118.3, 115.2, 114.5, 111.5, 54.5, 50.7, 44.5, 29.4, 20.2, 13.9. IR (KBr): 1606, 1585, 1562, 1441, 1426, 1397, 1368, 1300, 1253, 1226, 1060, 1016, 810, 775 cm^−1^. HRMS (ESI) *m*/*z* calcd for [C_30_H_35_N_2_S]^+^ (M^+^): 455.252; found: 455, 252.

#### Preparation of the nanoemulsions

##### Standard nanoemulsions

A stock solution of Lipoid® E80 in oil was obtained by dispersion of Lipoid® E80 (120 mg) in Miglyol® 840 (2 g). In order to obtain a complete dispersion, the mixture was heated to 70 °C. The dye (1.2 mg) was added to the stock solution (423 μL) in order to obtain 5 mM concentration. After 10 min sonication in an ultrasonic bath heated to 70 °C, a purple dispersion was obtained. The aqueous phase was prepared by dispersion of Tween® 80 (125 mg) in 4 mL of Milli-Q. An emulsion was then obtained by phase inversion (800 mg of aqueous phase (Milli-Q/Tween® 80) was added to 200 mg of oily phase) followed by homogenization during 7 min sonication in an ice bath using a Sonic Vibra-Cell VC 250 set at 70% and output 7. A milky droplet suspension was obtained.

##### Stealth nanoemulsions

To prepare stealth NEs incorporating the dye, 15 mg of PEGylated phospholipids (PE 18:0/18:0-PEG 2000) were added to the oily phase before homogenisation. For *in vitro* and *in vivo* experiments, the formulations’ pH was adjusted close to the physiological value (pH: 7.2) using sodium hydroxide 0.1 N. Glycerol at 2% was added to adjust the osmolality at 300 mOsm kg^−1^. The NE was visually inspected for eventual creaming, phase separation and/or dye precipitation. After preparation and prior to use in imaging, the formulations were stored at 4 °C, away from light and a colloidal stability assay (over 12 months) was accomplished by visual observation, granulometric analysis and zeta potential measurements over time.

##### NE characterization

The droplet physical properties (sizes and polydispersity index) were measured using a dynamic light scattering (DLS) device from Malvern Instruments (Zetasizer Nano ZS). The droplets’ sizes were determined directly after formulation (dilution 1/1000). The mean size was determined with three independent measurements performed at 25 °C. The zeta potential was determined by using a Zetasizer device from Malvern Instruments after dilution (1/1000) using a folded capillary cell (DTS1070, Malvern Instruments). Droplet concentration was investigated using a NanoSight NS300 from Malvern Instruments and was determined with five independent measurements. Nanoparticle tracking analysis was performed at 22 °C on samples after dilution (1/250 000).

TEM was performed at the Bordeaux Imaging Center on a Hitachi H7650 linked to an Orius SC1000 11MPX (GATAN) camera run by a Digital Micrograph (GATAN). All formulations were diluted 1/50 and transferred to a carbon-coated copper grid. Negative staining was performed with uranyl acetate for 1 minute.

##### Determination of the dye loading in NEs

The dye concentration in the NE[D] suspensions was determined by lyophilisation of an aliquot of the oil droplet suspension and dissolving the residue in chloroform. Measurements of the absorbance give access to the initial concentration of the dye in NE[D]. This protocol ensures that the use of the Beer–Lambert law to derive the dye concentration is valid. It allows circumventing scattering or flattening phenomena that may cause unreliability when measurements are conducted on NE suspensions.

#### Photophysical studies

##### UV-Vis absorption and emission spectroscopy

All photophysical properties were investigated with freshly prepared air-equilibrated solutions at room temperature (293 K). UV/Vis absorption spectra were recorded with a Jasco V-670 spectrophotometer. Steady-state fluorescence measurements were performed on dilute solutions (optical density < 0.1) contained in standard 1 cm quartz cuvettes by using a Horiba (FluoroLog or FluoroMax) spectrometer in photon-counting mode. Fully corrected emission spectra were obtained for each compound at *λ*^ex^ = *λ*^abs^_max_ with an optical density at *λ*^ex^ of ≤0.1 to minimize internal absorption. Fluorescence quantum yields were measured according to literature procedures^38,39^ by using 4-(dicyanomethylene)-2-methyl-6-(4-dimethylaminostyryl)-4*H*-pyran (DCM) in EtOH (*Φ*_f_ = 0.437) and indocyanine green in DMSO (*Φ*_f_ = 0.11) as standards.

##### Two-photon absorption

Two-photon absorption cross-sections (*σ*_2_) were determined from the two-photon excited fluorescence action cross-section (*σ*_2_*Φ*_f_) and the fluorescence quantum yield (*Φ*_f_). TPEF cross-sections were measured relative to fluorescein in 0.01 M aqueous NaOH in the 690–1000 nm spectral range and relative to Nile Red in DMSO in the 1000–1160 nm spectral range, using the method described by Xu and Webb^40^ and the appropriate solvent-related refractive index corrections.^41^ Reference values between 700 and 715 nm for fluorescein were taken from the literature. The quadratic dependence of the fluorescence intensity on the excitation power was checked at all wavelengths. Measurements were conducted using an excitation source delivering fs pulses. This allows avoiding excited-state absorption during the pulse duration, a phenomenon that has been shown to lead to overestimated two-photon absorption cross-section values. To scan the 680–1080 nm range, a Nd:YVO_4_-pumped Ti:sapphire oscillator was used generating 140 fs pulses at a 80 MHz rate. To scan the 1000–1400 nm range, an OPO (PP-BBO) was added to the setup to collect and modulate the output signal of the Ti:sapphire oscillator. The excitation was focused into the cuvette through a microscope objective (10×, NA 0.25). The fluorescence was detected in epifluorescence mode *via* a dichroic mirror (Chroma 675dcxru) and a barrier filter (Chroma e650sp-2p) using a compact CCD spectrometer module BWTek BTC112E. Total fluorescence intensities were obtained by integrating the corrected emission. The experimental uncertainty of the absorption cross-section values determined from this method has been estimated to be ±10%.

#### Live cell multimodal optical imaging

The preparations of the A431 and U87 human cells for *in vitro* live cell imaging are described in the ESI.†

The concentrations of the NEs for the imaging on live A431 and U87 cells were set at 1/100 and 1/1000 of the nominal concentration, respectively. For co-localization experiments, we followed the staining procedure recommended by the MitoTracker and LysoTracker supplier (Molecular Probes). Thus the concentrations were set at 200 nM and 100 nM for MitoTracker and LysoTracker, respectively. A complete description of the conditions and the imaging procedure are given in the ESI.†

The confocal microscope used was a Leica TCS SP5 on an upright stand DM6000 (Leica Microsystems, Mannheim, Germany), equipped with the objective HCX APO L U-V-I 63.0× water NA 0.90. For confocal microscopy, the excitation source was a 561 nm laser and the photons were collected in the 650–800 nm (A431 cells) and 610–800 nm (U87 cells) wavelength range. The multiphoton microscopy was performed with a tuneable pulsed laser Mai Tai HP (Spectra-Physics, Irvine, USA), tuned at 820, 950 or 1020 nm). Photons were collected by an internal PMT in the 650–800 nm (A431 cells) and 610–800 nm (U87 cells) wavelength range. The Second Harmonic Generation (SHG) acquisitions were done with the excitation wavelength 896 nm in the transmitted light pathway, equipped with a short-pass filter (SP 680 nm) and a bandpass filter (448/20 nm). The system was used with a conventional scanner.

#### *In vivo* imaging

All animal procedures were performed in accordance with the Guidelines for Care and Use of Laboratory Animals of European community and approved by the Animal Ethics Committee of Bordeaux (C2EA 50) under agreement A50120196.

Murine prostate cancer cell line RM1, initially obtained from Dr T. C. Thompson (Baylor College of Medicine, Houston, TX, USA), was genetically engineered for constitutive expression of firefly luciferase (RM1-CMV/Fluc) as previously described^15^ and was maintained in Dulbecco's modified Eagle's medium (Invitrogen, Carlsbad, CA, USA) supplemented with 10% fetal bovine serum (Invitrogen), 1% antimycotic–antibiotic mix (PSA, Invitrogen) and blasticidin (10 μg mL^−1^, Euromedex, Souffelweyersheim, France). The cell line was maintained in a humidified 5% CO_2_ incubator at 37 °C.

Black-6 albino mice were anesthetized with 2% isoflurane (Belamont, Nicholas Piramal Ltd, London, UK) in air. A subcutaneous tumour was generated on the right leg (2 × 10^6^ cells/100 μL). Prior to the imaging session, the regions of the mice to be imaged were shaved with clippers and a depilatory cream. Four days after injection of RM1 cells, the formulations (20 μL diluted in 80 μL of 5% glucose) were administered intravenously in the tail vein (*N* = 3 per condition).

Fluorescence reflectance imaging (FRI) was performed at Vivoptic (UMS 3767-University of Bordeaux) using a Lumina LT (PerkinElmer Inc., Boston, MA, USA). Excitation was performed at 535 nm and emission was detected with a Cy5.5 filter (695–770 nm).

## Conflicts of interest

There are no conflicts to declare.

## Supplementary Material

NA-002-C9NA00710E-s001

NA-002-C9NA00710E-s002

NA-002-C9NA00710E-s003

NA-002-C9NA00710E-s004
