# Supplementary material for: Multimodal optical contrast agents as new tools for monitoring and tuning nanoemulsion internalisation into cancer cells. From live cell imaging to in vivo imaging of tumours
Source: Nanoscale Adv. 2020 Feb 27;2(4):1590–602. doi: 10.1039/c9na00710e (PMC9416932; doi:10.1039/c9na00710e)
Supplement: NA-002-C9NA00710E-s003 [file NA-002-C9NA00710E-s003.pdf]

Electronic Supplementary Material (ESI) for Nanoscale Advances.
This journal is © The Royal Society of Chemistry 2020

Supporting Information

Content

1 Synthesis of intermediate compounds 2

2 Characterisation of the bare and pegylated NEs 3

3 Photophysical properties 4

4 Cytotoxicity studies of the NEs 7

5 Live cells multimodal optical imaging 7

5.1 Monitoring of NEs uptake in U87 cells by confocal and two-photon imaging 8

5.2 Monitoring of endocytosis of NE[D2]-PEG in U87 cells by SHG 8

5.3 Co-localisation experiments in U87 cells: 9

5.4 Ex-vivo fluorescence imaging 14

1 Synthesis of intermediate compounds

4-iodo-N,N-dibutylaniline (1)[1]: A solution of $NaHCO_3$ (2.68 g, 32.01 mmol) in 48 mL of water was added to a solution of N,N-dibutylaniline (4.00 g, 19.47 mmol) in 15 mL of CH_2Cl_2. The mixture was cooled down to 0°C and a solution of iodine (5.10 g, 20.37 mmol) in 225 mL de CH_2Cl_2 was added dropwise, during 1 hour. The mixture was kept under stirring overnight at room temperature. The reaction was quenched with $Na_2S_2O_3$ (100 mL), and the organic phase was washed with Na2S2O3 (2*100 mL), water and dried over anhydrous Na_2SO_4. After filtration, the solvent was evaporated under vacuum and the residue was purified by silica-gel column chromatography using petroleum ether to afford 1 as a pale yellow oil (4.3 g, 67%).^{1}H NMR ($CDCl_3$, 300 MHz): δ 7.40 (d, J = 9.1 Hz, 2H), 6.40 (d, J = 9.1 Hz, 2H), 3.21 (t, J = 7.6 Hz, 4H), 1.53 (quint, J = 7.5 Hz, 4H), 1.33 (sext, J = 7.4 Hz, 4H), 0.94 (t, J = 7.3 Hz, 4H).

5-(4-(dibutylaminophenyl)-2-thiophenecarboxaldehyde (2a)[2,3]: To an oven dried round bottom flask cooled under argon, were added 4-iodo-N,N-dibutylaniline (1.4 g, 4.22 mmol), 5-formyl-2-thiopheneboronic acid (1.05 g, 6.75 mmol), K_2CO_3 (1.45 g, 10.55 mmol), Pd(dppf)Cl2 (172 mg, 0.21 mmol) in the solvent consisting of toluene (10 mL) and methanol (10 mL). The mixture was stirred at 80 °C for 12 h. After cooling down, the mixture was filtered through a pad of celite, rinsed with dichloromethane then the filtrate was concentrated under vacuum. The residue was purified by silica-gel column chromatography using a 1/1 dichloromethane/petroleum ether mixture and yielded 769 mg (60 %) of the desired compound 2 as a yellow-green solid. ^{1}H NMR (300 MHz, $CDCl_3$) δ 9.80 (s, 1H) 7.66 (d, J = 4 Hz, 1H), 7.52 (d, J = 8.9 Hz, 2H), 7.21 (d, J = 4.0 Hz, 1H), 6.63 (d, J = 8.9 Hz, 2H), 3.31 (t, J = 7.6 Hz, 4H), 1.59 (quint, J = 7.5 Hz, 4H), 1.37 (sext, J = 7.4 Hz, 4H), 0.96 (t, J = 7.3 Hz, 6H).IR (KBr): 2955, 1660, 1603, 1445, 1234, 1060, 790 cm^{-1}.

4-(Diphenylphosphinoylmethyl)quinoline (3q)[4]: 4-methylquinoline (3 g, 20.9 mmol, 2.7 mL) was dissolved in 50 mL of anhydrous THF. The solution was cooled to -78°C and 1 equivalent of LDA (prepared in situ with 20.9 mmol, 9.4 mL of 2.21 M of BuLi in hexane added to 20.9 mmol, 2.11 g (2.9 mL) of anhydrous diisopropylamine in 15 mL of anhydrous THF) was added dropwise. The orange solution was maintained at -78°C for 2 h. Then chlorodiphenylphosphine (5.07 g, 22.9 mmol, 4.2 mL) was added. After stirring at -78°C for 30 minutes, the reaction mixture was slowly allowed to warm to room temperature. The solvent was concentrated under vacuum, and the residue was extracted with dichloromethane and the organic phase was washed with water, dried over anhydrous Na_2SO_4, filtrated and concentrated under vacuum. The oily residue was dissolved in 90 mL of toluene and the solution was refluxed under air bubbling for 16 h. After cooling down to room temperature, the mixture was filtered and the solid was collected and rinsed with toluene to afford 3 as a white solid (4 g, yield 57 %).

^{1}H NMR ($CDCl_3$, 300 MHz): δ 8.68 (d, J = 4.5 Hz, 1H), 8.05 (d, J = 8.3 Hz, 1H), 7.93 (d, J = 8.3 Hz, 1H), 7.62-7.73 (m, 5H), 7.39-7.54 (m, 7H), 7.14-7.17 (m, 1H), 4.10 (d, J = 14.9 Hz, 2H). 31P NMR ($CDCl_3$, 121.4 MHz): δ 28.7.

2 Characterisation of the bare and pegylated NEs

Table S1: Morphological characteristics of the dye-loaded NEs (bare NE made from lecithins and pegylated NE made from DSPE-PEG2000)

	Diameter [a)] nm	PdI [b)]	Zeta pot. mV
**NE[D1]**	143	0,04	+16.2
**NE[D2]**	151	0.12	+15.9
**NE[D1]-PEG**	180	0.13	-43.2
**NE[D2]-PEG**	162	0.10	-57.6

[a)] Droplet diameter determined by DLS, [b)] polydispersity index

Table S2. Evolution of the characteristics of the control and dye-loaded NEs over time.

	Day(s)	0	7	60	1 year
**NE**-control	Diameter (nm)	151.1 ± 1.5	160.6 ± 1.5	170.3 ± 1.4	205.7± 1.1
	PdI	0.104	0.125	0.159	0.174
	Zeta pot. (mV)	-31.6 ± 8.10	-33.8 ± 5.26	-36.2 ± 6.32	-31.8 ± 6.03
**NE[D2]**	Diameter (nm)	150.7 ± 0.6	163.2 ± 1.5	169.5 ± 0.1	180.3 ± 0.8
	PdI	0.123	0.119	0.122	0.165
	Zeta pot. (mV)	15.9 ± 7.08	15.6 ± 7.81	13.7 ± 5.97	13.9 ± 5.86

Table S3: Comparison of the absorption and emission properties of the bare and pegylated dye-loaded NEs.

Dye	λ_{max}^{exc} nm	λ_{max}^{em} nm
**NE[D1]**	495	707
**NE[D1]-PEG**	490	705
**NE[D2]**	540	742
**NE[D2]-PEG**	540	735

3 Photophysical properties

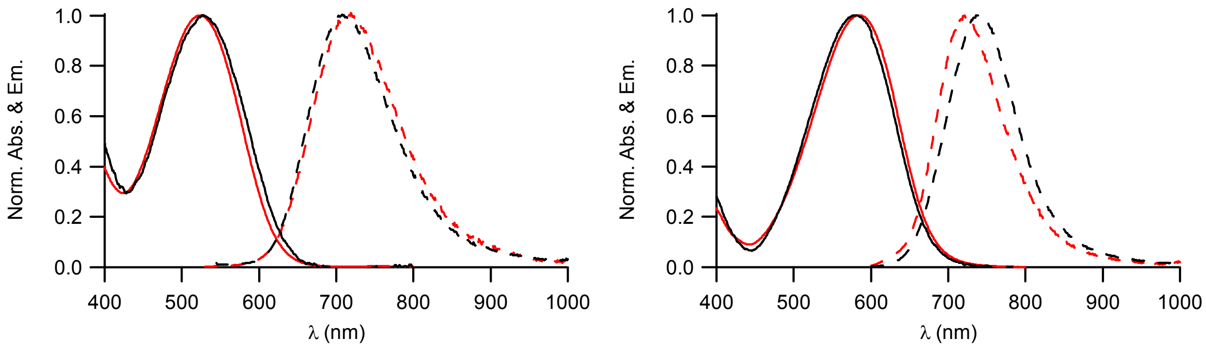

Figure S1. Absorption and emission spectra of the dyes **D1** (left) and **D2** (right) in solution in toluene (black) and in micellar water (red) (SDS/butanol/water, 6: 5: 89 wt %).

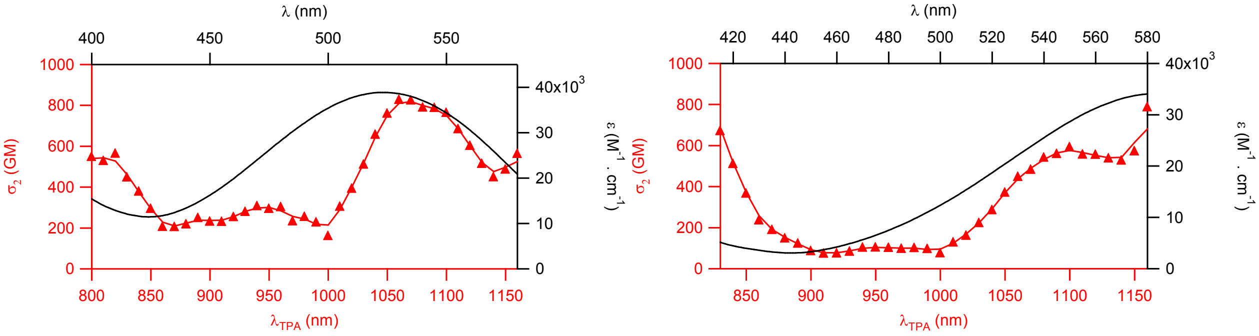

Figure S2: Comparison of rescaled 1PA (black) and 2PA (red) of dyes **D1** (left) and **D2** (right) in micellar water (SDS/butanol/water, 6: 5: 89 wt %).

Figure S3: Plots of the two-photon excited fluorescence signal versus laser power for dye **D2** at various wavelengths in the 800-850 nm range.

@800 nm

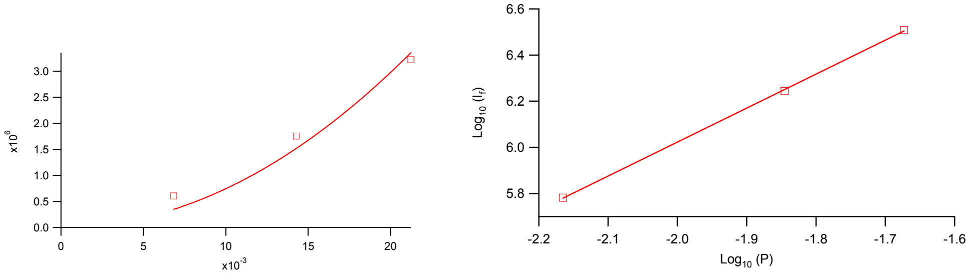

slope 1.5

@820 nm

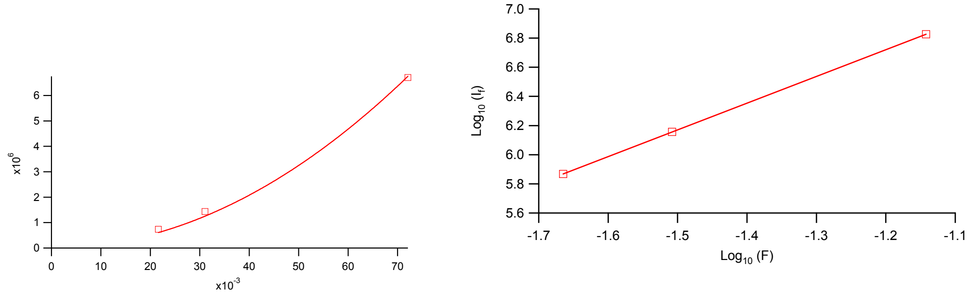

slope 1.8

@830 nm

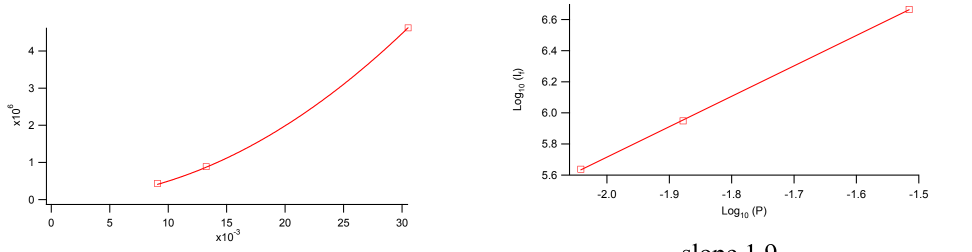

slope 1.9

@840 nm

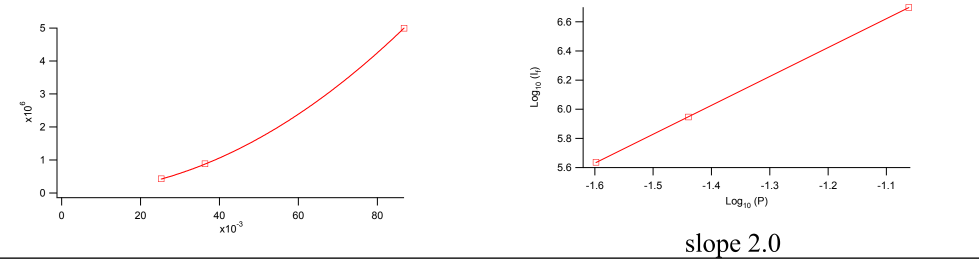

slope 2.0

@850 nm

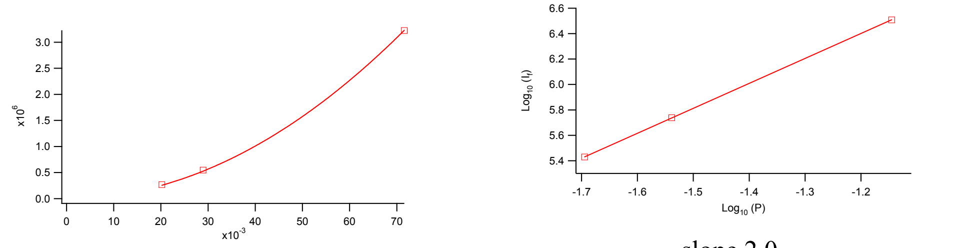

slope 2.0

Figure S54. Plots of the two-photon excited fluorescence signal versus laser power for dye **D1** at 800 and 820 nm.

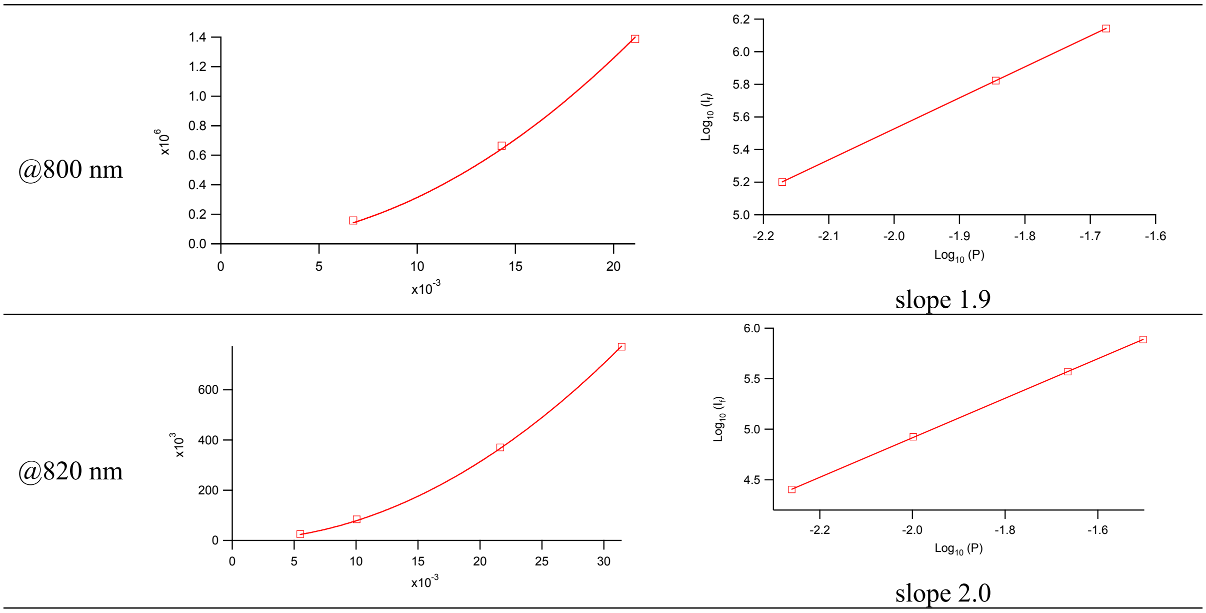

4 Cytotoxicity studies of the NEs

U87-CMV/LucF cell line (U87 MG, human glioblastoma, ATCC, Manassas, MD, USA) was generated as previously reported[5]. Cells were grown in Dulbecco's modified Eagle's Medium without phenol-red (DMEM, Dutscher, France) supplemented with 10 % v/v foetal bovine serum (FBS, Invitrogen, Carlsbad, CA, USA), 1 % V/V antibiotic-antimycotic mix (PSA, Invitrogen), 1% V/V non-essential amino-acid (MEM NEAA, Invitrogen) and 50 µg/mL Hygromycin B (Euromedex, Souffelweyersheim, France). Cell line was maintained in a humidified incubator at 37°C and 5% CO_2. For cytotoxicity studies, the cells were plated in 24-well plates and were incubated at 37 °C, 5 % CO_2 in water saturated atmosphere for one day. The next day the cells were incubated with 5µL to 1µL of a 1/10 diluted solution in water of **NE[D1], NE[D1]-PEG, NE[D2]** or **NE[D2]-PEG**, at 37 °C, 5 % CO_2 for 2 days. The cell viability was followed by bioluminescence of the cells. Bioluminescence images were taken 8 min after addition of luciferin as previously described.[5] Results are presented on Figure S5.

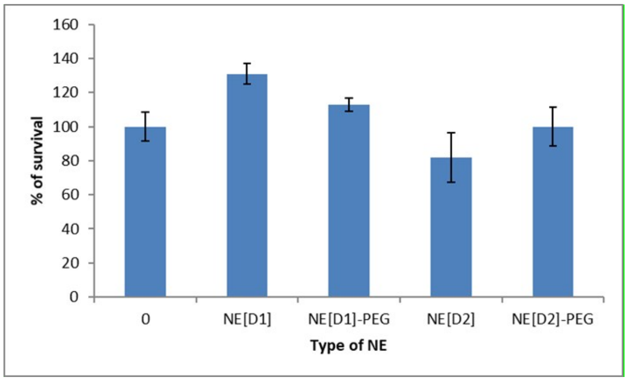

Figure S5. Survival of U87-CMV/LucF cells in the presence of 1/1000 final dilution of NEs (mean +/- standard deviation for n = 3).

5 Live cells multimodal optical imaging

Human cells A431 were grown in Dulbecco's modified Eagle medium without phenol-red (DMEM PAN Biotech P04-03591) supplemented with 10 % v/v foetal calf serum (FCS-Dominique Dutscher, 500105), 1 % V/V penicillin/streptomycin (Dominique Dutscher, P06-07100) in 25 mL flasks (Falcon, 353082) at 37 °C, 5 % CO_2. They were kept below 90 % confluence, at which cells were washed in sterile filtered PBS (PAN Biotech, P04-36500), detached in a small volume of trypsin (PAN Biotech, P10-021100), washed in full warm medium and split 1/10 in a new flask. For imaging, 90 % confluent cells were similarly detached with trypsin and diluted 1/10 in full warm medium. 18 mm-diameter round#1.5 coverslips (Thermo Scientific, DV40008) were placed in a 12-well plate (Cellstar, 665180); 1 mL of full medium was added on each coverslip followed by the addition of a volume of diluted cells in

order to get 60 000 cells per well (100 µL on average). Cells were carefully homogenized by tilting the 12-well plate, and left at 37 °C, 5 % CO_2 for two days. Before imaging, the cells were incubated with 10 µL of the solution of **NE[D2]** at 37 °C, 5 % CO_2 for 1 h 45, then the medium was removed and the cells were washed once with warm PBS, then the wells were fill with 1 mL of warm PBS. Afterward, the live cells were imaged in a petri dish maintained at 37 °C and 5 % CO_2, using a temperature control device (Tokai Hit, INUBG2-GSU).

For imaging on U87-CMV/LucF cells (100 000 cells/2 mL) cells were plated in 6-well plates and Petri dishes of 35 mm diameter. Plates and Petri Dishes were incubated at 37 °C, 5 % CO_2 in water saturated atmosphere for one day. Before imaging, the cells were incubated with 20 µL of a 1/10 diluted solution in water of **NE[D1]-PEG** or **NE[D2]-PEG**, at 37 °C, 5 % CO_2 for 45 min, then the medium was removed and the cells washed once with warm PBS, then the wells were filled with 1 mL of warm PBS. The cellular uptake of the **NE[D1 or D2]-PEG** was followed on Petri dishes maintained at 37 °C and 5 % CO_2, using a temperature control device (Tokai Hit, INUBG2-GSU).

5.1 *Monitoring of NEs uptake in U87 cells by confocal and two-photon imaging*

The first image of the monitoring of the uptake of the **NE[D1]-PEG** by U87 cells was acquired 2 min after the addition of **NE[D1]-PEG** in cells imaging medium for 47 min with a time-lapse of 1.6 min between each image. For each snapshot, the bright-field, the two-photon imaging (λ^{exc} = 820 nm, 950 nm and 1020 nm) and the confocal imaging (λ^{exc} = 561 nm) were acquired (Movie S1).

The first image of the following of the uptake of the **NE[D2]-PEG** by U87 cells was acquired 7 min after the addition of **NE[D2]-PEG** in cells imaging medium for 47 min with a time-lapse of 1.5 min between each images. For each snapshot, the bright-field, the two-photon imaging (λ^{exc} = 820 nm, and 1020 nm) and the confocal imaging (λ^{exc} = 561 nm) were acquired (Movie S2).

5.2 *Monitoring of endocytosis of NE[D2]-PEG in U87 cells by SHG*

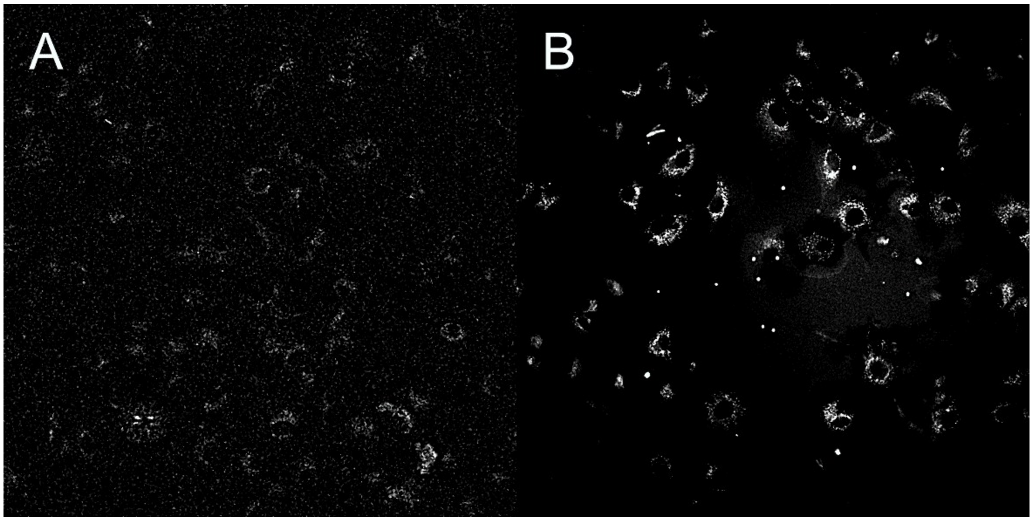

Figure S6. SHG imaging on U87 cells incubated with **NE[D2]-PEG** for 1 h (A) and 2 h (B).

In order to confirm the origin of the fluorescence signal obtained by two-photon imaging we recorded the emission spectrum of the two-photon imaging of U87 cells at the end of the uptake experiment (i.e. 50 min of incubation time with NEs). The resulting emission spectra are shown in Figure S7 and clearly evidenced that the fluorescence signal is only due to the **D1** and **D2** dyes and not from auto-fluorescence background.

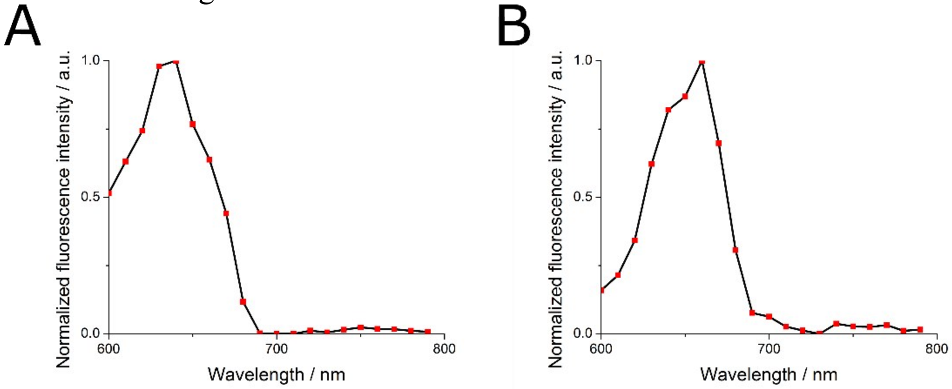

Figure S7. Normalized emission spectra (λ^{2PE} = 820 nm) recorded in U87 cells incubated for 50 min with **NE[D1]-PEG** (A) and **NE[D2]-PEG** (B).

5.3 *Co-localisation experiments in U87 cells:*

For co-localization experiments, U87 cells were incubated with **NE[D1]-PEG** or **NE[D2]-PEG** and MitoTracker Green (final concentration = 200 nM) or LysoTracker Blue DND-22 (final concentration = 100 nM) at 37 °C for 45 min. Then, the cells were washed once with warm PBS then imaged directly into warm PBS. For each sample, Z-stack of bright-field, two-photon imaging and confocal imaging were acquired.

The Pearson's correlation coefficient was calculated on ROIs using the same plane of the confocal imaging of NEs and commercial probes (MitoTracker or LysoTracker) and using the FIJI software (plugin Coloc2). For each sample, we measured the co-localization of the NEs with commercial probes on 5 cells then on the whole image. The resulting averaged Pearson's R value are: 0.08 ± 0.06 (**NE[D1]-PEG** with MitoTracker), 0.59 ± 0.08 (**NE[D1]-PEG** with Lysotracker), 0.35 ± 0.07 (**NE[D2]-PEG** with MitoTracker), 0.57 ± 0.13 (**NE[D2]-PEG** with LysoTracker). Pearson's correlation coefficient is used to measure the co-localization (its value ranging from -1 to 1), where 1 corresponds to full co-localization while 0 corresponds to no co-localization. Thus, the **NE[D1]-PEG** does not stain mitochondria contrary to **NE[D2]-PEG** while both NEs stain lysosomes to the same extent.

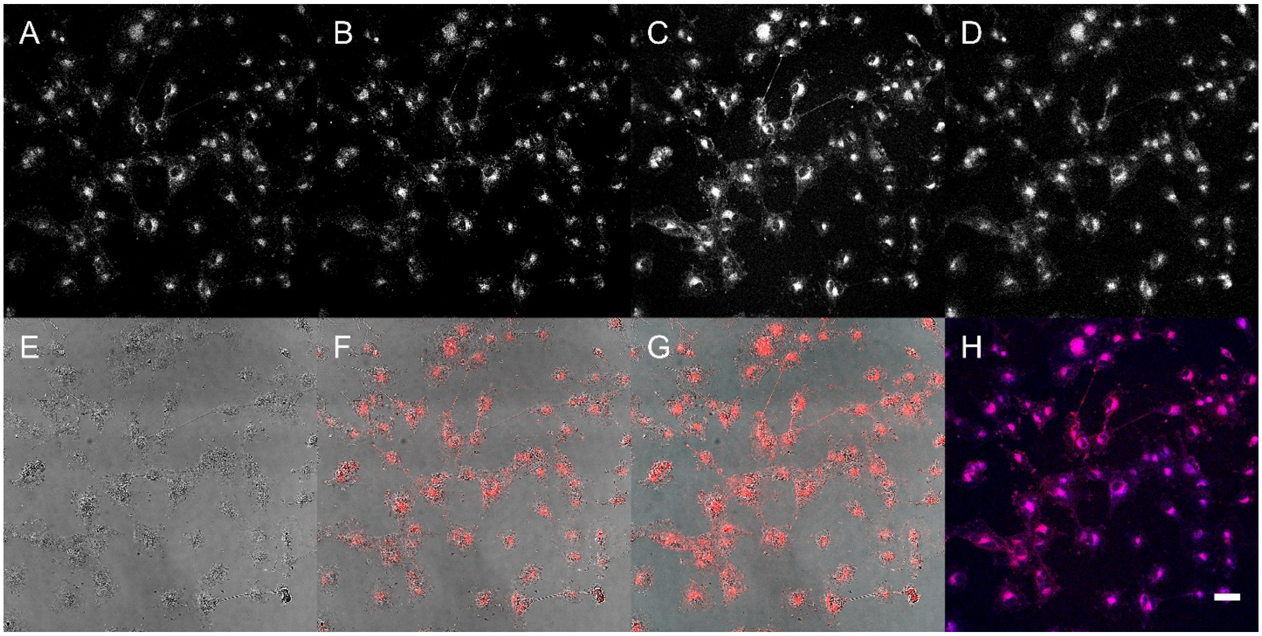

Figure S8. Stacked images of U87 cells incubated with LysoTracker and **NE[D1]-PEG**. (A) Two-photon imaging (λ^{exc} = 820 nm). (B) Two-photon imaging (λ^{exc} = 1020 nm). (C) **NE[D1]-PEG** confocal imaging (λ^{exc} = 561 nm). (D) LysoTracker confocal imaging (λ^{exc} = 405 nm). (E) Bright-field. (F) Merged bright-field with two-photon imaging (λ^{exc} = 1020 nm). (G) Merged bright-field with confocal imaging (λ^{exc} = 561 nm). (H) Merged **NE[D1]-PEG** (λ^{exc} = 561 nm, red channel) with LysoTracker (λ^{exc} = 405 nm, blue channel) confocal imaging. Fluorescence is collected in 610-800 nm range for A-C and in 415-480 nm range for D. Scale bar = 20 µm.

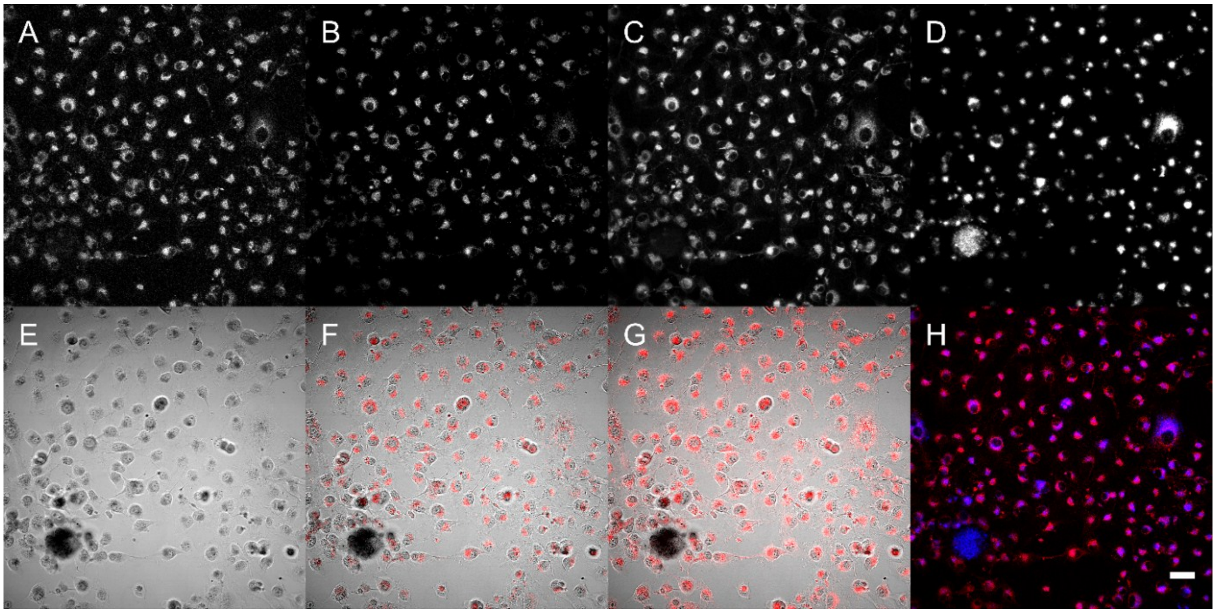

Figure S9. Stacked images of U87 cells incubated with LysoTracker and **NE[D2]-PEG**. (A) Two-photon imaging (λ^{exc} = 820 nm). (B) Two-photon imaging (λ^{exc} = 1020 nm). (C) **NE[D2]-PEG** confocal imaging (λ^{exc} = 561 nm). (D) LysoTracker confocal imaging (λ^{exc} = 405 nm). (E) Bright-field. (F) Merged bright-field with two-photon imaging (λ^{exc} = 1020 nm). (G) Merged bright-field with confocal imaging (λ^{exc} = 561 nm). (H) Merged **NE[D2]-PEG** (λ^{exc} = 561 nm, red channel) with LysoTracker (λ^{exc} = 405 nm, blue channel) confocal imaging. Fluorescence is collected in 610-800 nm range for A-C and in 415-480 nm range for D. Scale bar = 20 μm.

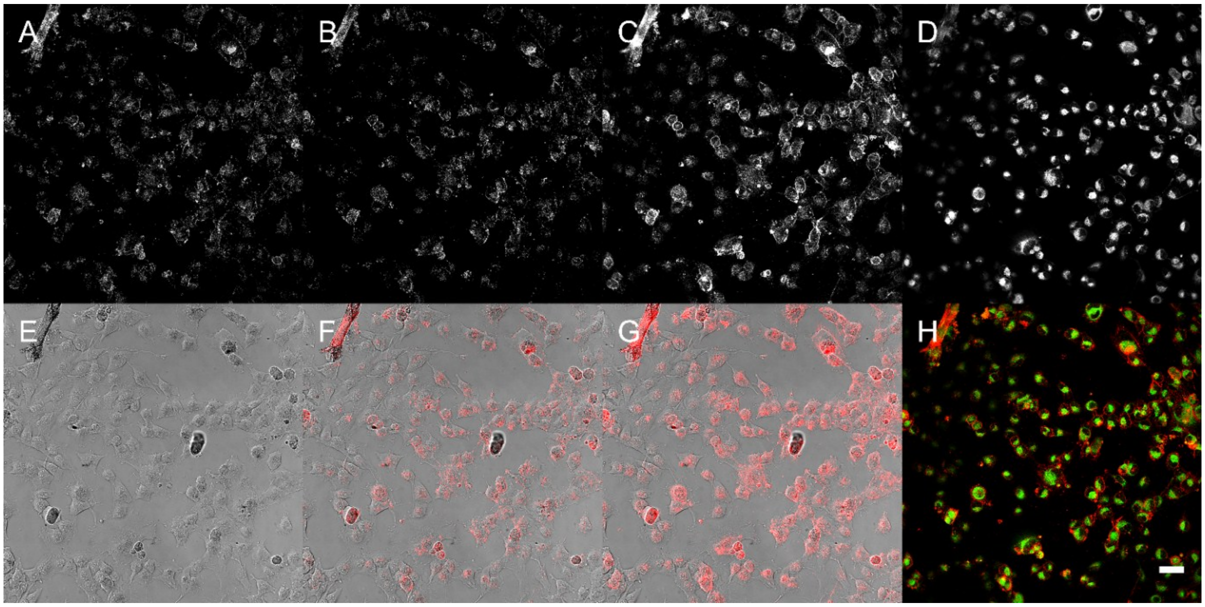

Figure S10. Stacked images of U87 cells incubated with MitoTracker and **NE[D1]-PEG**. (A) Two-photon imaging (λ^{exc} = 820 nm). (B) Two-photon imaging (λ^{exc} = 1020 nm). (C) **NE[D1]-PEG** confocal imaging (λ^{exc} = 561 nm). (D) MitoTracker confocal imaging (λ^{exc} = 488 nm). (E) Bright-field. (F) Merged bright-field with two-photon imaging (λ^{exc} = 1020 nm). (G) Merged bright-field with confocal imaging (λ^{exc} = 561 nm). (H) Merged **NE[D1]-PEG** (λ^{exc} = 561 nm, red channel) with MitoTracker (λ^{exc} = 488 nm, green channel) confocal imaging. Fluorescence is collected in 610-800 nm range for A-C and in 500-550 nm range for D. Scale bar = 20 μm.

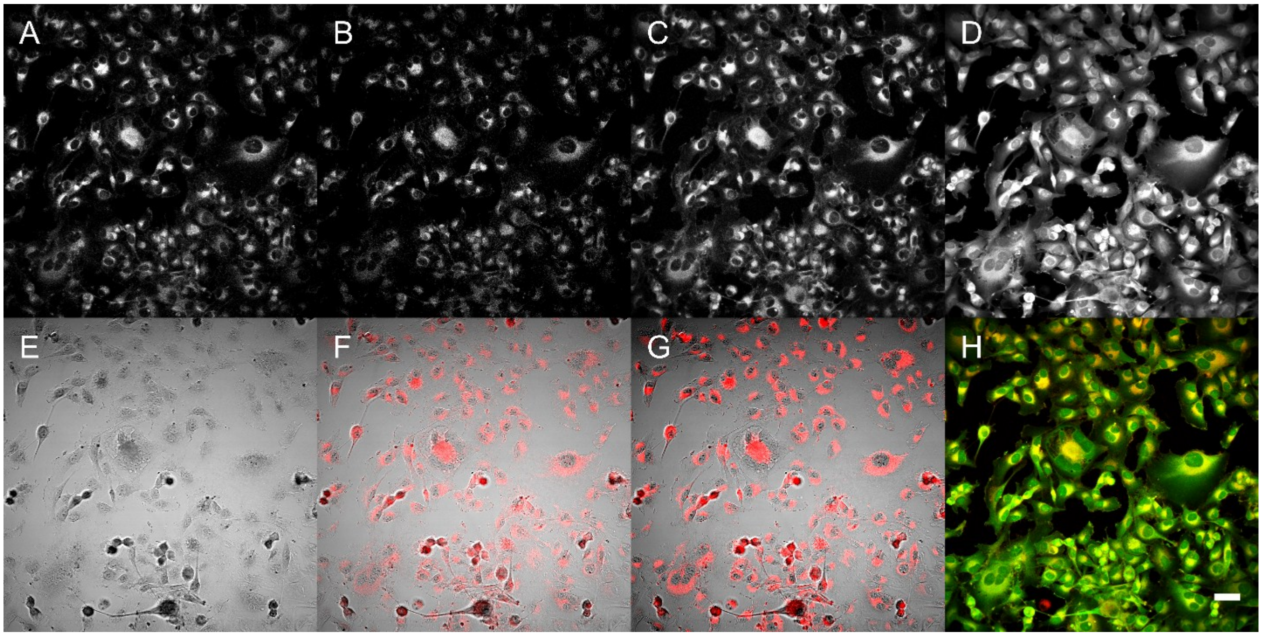

Figure S11. Stacked images of U87 cells incubated with MitoTracker and **NE[D2]-PEG**. (A) Two-photon imaging (λ^{exc} = 820 nm). (B) Two-photon imaging (λ^{exc} = 1020 nm). (C) **NE[D2]-PEG** confocal imaging (λ^{exc} = 561 nm). (D) MitoTracker confocal imaging (λ^{exc} = 488 nm). (E) Bright-field. (F) Merged bright-field with two-photon imaging (λ^{exc} = 1020 nm). (G) Merged bright-field with confocal imaging (λ^{exc} = 561 nm). (H) Merged **NE[D2]-PEG** (λ^{exc} = 561 nm, red channel) with MitoTracker (λ^{exc} = 488 nm, green channel) confocal imaging. Fluorescence is collected in 610-800 nm range for A-C and in 500-550 nm range for D. Scale bar = 20 µm.

5.4 *Ex-vivo fluorescence imaging*

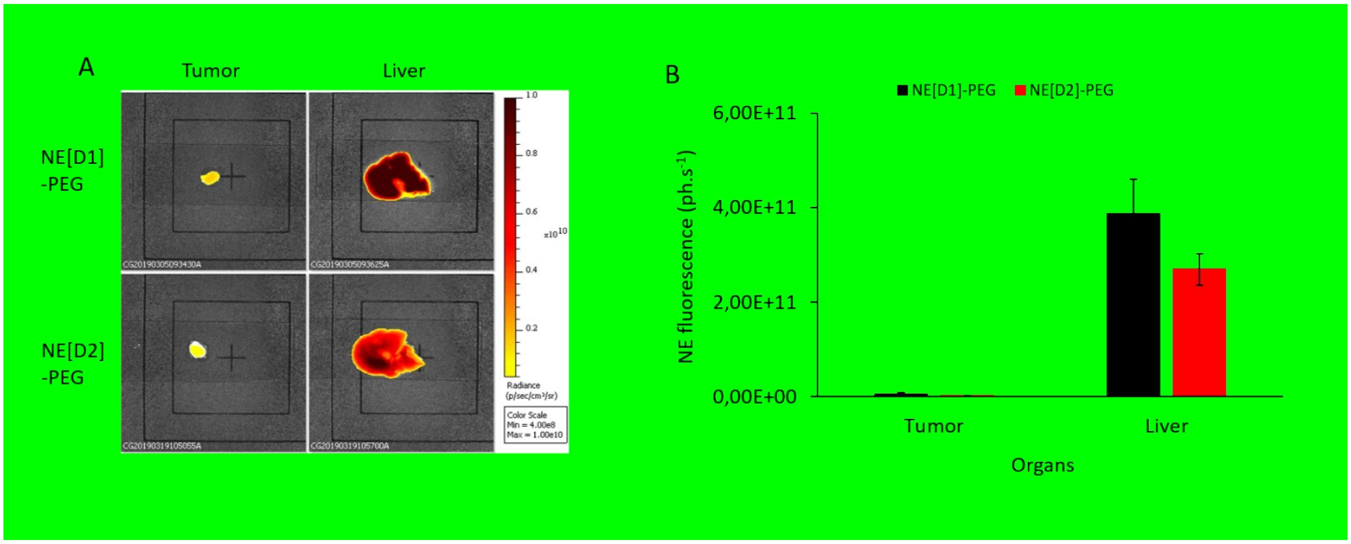

Figure S12. Fluorescence signal ex-vivo in subcutaneous tumours and liver 24 hours after NEs injection (A) Representative images of fluorescence reflectance signals in tumour and liver of mouse injected with NE[D1]-PEG or NE[D2]-PEG injection. (B) Quantification of fluorescence signal in tumour and liver 24 hours after NE[D1]-PEG (black) or NE[D2]-PEG (red) injection (Mean +/- standard deviation for n = 3).

References:

1 S. Rondeau-Gagné, C. Curutchet, F. Grenier, G. D. Scholes, J.-F. Morin, *Tetrahedron*, 2010, **66**, 4230-4242.

2 Z.-Y. Hu, A. Fort, M. Barzoukas, A. K.-Y. Jen, S. Barlow, S. R. Marder, *J. Phys. Chem. B*, 2004, **108**, 8626-8630.

3 S. Zhang, A. Islam, X. Yang, C. Qin, K. Zhang, Y. Numata, H. Chen, L. Han, *J. Mater. Chem. A*, 2013, **1**, 4812-4819.

4 J. Daniel, C. Mastrodonato, A. Sourdon, G. Clermont, J. M. Vabre, B. Goudeau, H. Voldoire, S. Arbault, O. Mongin, M. Blanchard-Desce, *Chem. Commun.*, 2015, **51**, 15245-15248.

5 P.-Y. Fortin, C. Genevois, A. Koenig, E. Heinrich, I. Texier F. Couillaud. *J. Biomed. Opt.*, 2012, **17**, 126004.
